# Explainable ensemble transfer learning for skin lesion classification with multi-method explainability validation

**DOI:** 10.3389/fpubh.2026.1847649

**Published:** 2026-09-03

**Authors:** Nemili Sravani, Srinivas Koppu

**Affiliations:** School of Computer Science Engineering and Information Systems, Vellore Institute of Technology, Vellore, India

**Keywords:** ensemble learning, explainable artificial intelligence, Grad-CAM, HAM10000, lime, occlusion sensitivity, skin lesion classification, transfer learning

## Abstract

**Introduction:**

Skin cancer is among the most prevalent and life-threatening malignancies worldwide. Early and accurate detection significantly improves therapeutic outcomes. Automated classification of dermoscopic skin lesions remains challenging due to class imbalance, inter-class visual similarity, and lack of interpretable predictions. This study proposes an accurate and interpretable weighted soft voting ensemble for multi-class skin lesion classification.

**Methods:**

A weighted soft voting ensemble of three heterogeneous deep learning architectures, EfficientNet-B3, ResNet101, and DenseNet201, pretrained on ImageNet and fine-tuned on the HAM10000 dataset comprising 10,015 dermoscopic images across seven diagnostic categories (AKIEC, BCC, BKL, DF, MEL, NV, and VASC), was developed. The framework integrates complementary feature extraction based on compound scaling, residual learning, and dense connectivity, along with class-specific augmentation using differentiated transformation parameters for the minority classes AKIEC, DF, and VASC. Explainability was evaluated using three complementary XAI methods, Grad-CAM, LIME, and Occlusion Sensitivity Analysis (OSA), using the Trustworthiness Metric for AI (TMAI) to quantitatively assess explanation quality. Generalizability was further assessed on the ISIC 2019 benchmark dataset using a separately trained model comprising 25,331 images across eight categories.

**Results:**

The proposed framework achieved an accuracy of 96.37%, precision of 96.25%, recall of 96.37%, F1-score of 96.23%, and macro-average AUC of 0.983 on HAM10000. The model achieved perfect classification for BCC (100%, 35/35; 95% CI: 90.1%–100.0%) and VASC (100%, 13/13; 95% CI: 77.2%–100.0%), and achieved 99.77% accuracy for MEL. The proposed ensemble surpassed the best individual model, EfficientNet-B3 (95.38%), by 0.99 percentage points and exceeded the clinically recommended AUC threshold of 0.95. Three-fold cross-validation confirmed the consistency of the reported results, achieving a mean accuracy of 95.56% ± 0.32% across all folds. On the ISIC 2019 dataset, the separately trained model achieved an accuracy of 91.47%, F1-score of 91.28%, and AUC of 0.9818 without any architectural modifications.

**Discussion:**

The proposed heterogeneous weighted ensemble demonstrates high classification performance and consistent generalizability across independent dermoscopic datasets. Quantitative evaluation using TMAI and complementary XAI methods provides additional evidence that the model's predictions are grounded in clinically relevant lesion regions rather than background artefacts, supporting the potential of the framework for accurate and interpretable automated skin lesion classification.

## 1 Introduction

Skin diseases are a major public health challenge, affecting nearly one-quarter of the worldwide population and ranking among the top causes of non-fatal disease. The World Health Organization (WHO) reports that about one in three people has a skin disease. This shows how common and serious these conditions are for global healthcare. Skin diseases range from mild infections and rashes to common inflammatory conditions such as eczema and psoriasis, and to life-threatening malignancies including melanoma (1). Skin diseases include inflammation, infections, autoimmune disorders, and skin cancers. Among all of these skin diseases, skin cancer is considered one of the most common cancers worldwide; melanoma can be dangerous if it is not detected and treated early. The number of skin problems is increasing day by day due to various factors such as aging populations, higher UV exposure, and changes in environmental conditions. Skin diseases not only affect a patient's physical appearance, but also deeply impact a person's psychological well-being, emotions, and daily quality of life. Timely and accurate diagnosis of skin diseases plays an important role in providing effective treatment and patient outcomes (2). In particular, early detection of malignant lesions increases the chances of survival. The rapid advancement of Artificial Intelligence (AI) and Deep Learning (DL) methods has brought major changes to medical image analysis, helping improve clinical decision-making in dermatology diagnosis (3). Convolutional neural networks (CNNs) have achieved dermatologist-level, or sometimes even better, performance by automatically extracting hierarchical features from dermoscopic and clinical images, achieving strong performance metrics and outperforming expert dermatologists in certain diagnostic tasks. These AI models are good at spotting complex patterns and subtle morphological features in dermoscopic images that may remain undetected by the human eye, improving diagnostic accuracy and reliability across clinical environments (4). Implementing AI models in dermatological practice is not merely about automating detection and classification. These systems serve as clinical decision support tools that provide dermatologists with comprehensive skin lesion characteristics and risk score estimates. Recent studies have demonstrated that DL models can process multimodal data, including patient demographics, medical history, and skin images, to deliver accurate diagnostic results. This augmented intelligence approach combines clinical expertise and AI models to improve diagnostic speed and accuracy. Developing an efficient AI model for skin disease diagnosis is limited by a lack of access to large, labeled medical image datasets. To overcome such limitations, Transfer Learning (TL) is considered an effective technique in medical image analysis. This approach uses neural networks pre-trained on large datasets such as ImageNet and then fine-tuned to perform specialized tasks on medical images, such as classification and segmentation (5). In dermatology, TL-based models like ResNet, EfficientNet, and DenseNet achieve strong results, especially for classifying different types of skin conditions with high accuracy. These models show how TL overcomes limited data and improves model performance in real-time dermatology applications.

Deep learning models provide optimal accuracy in dermatology, but they lack transparency, referred to as their black-box nature, which makes it difficult for them to be adopted in clinical practice and receive regulatory approval (6). In healthcare applications, interpretability is not just a technical choice; it is essential for doctors and AI systems to trust each other, ensure patient safety, and make medical decisions ethically and legally. AI systems must not only make correct predictions but also provide clear, understandable explanations that align with dermatological reasoning and can be reviewed by clinical experts. XAI techniques are important for explaining how complex neural networks reach final decisions in skin disease diagnosis, making their results easier for doctors to understand and trust (7). XAI techniques like Grad-CAM, LIME, and OSA offer visual explanations that highlight specific regions of skin lesions or other important features influencing a model's decision. These techniques help clinicians verify that models focus on clinically relevant skin areas rather than unrelated patterns or image noise. The combination of TL and XAI techniques presents a promising framework for developing clinically deployable systems that leverage pretrained models for robust feature representations while providing transparent insights into their decision-making processes, supporting clinical validation and trust among healthcare practitioners (8).

Despite the growing use of XAI in dermatology, a reliable and trustworthy metric is still lacking to evaluate whether these explanations focus on the correct lesion regions or background areas. TMAI fills this gap by automatically segmenting lesion regions and measuring the explanation's intensity within the lesion boundary, generating an objective trustworthiness score for each XAI model. Additionally ensemble models combining predictions from multiple different architectures have proven that the performance is better than individual models by identifying complementary features but their explainability has so far been rarely validated on the HAM10000 benchmark dataset containing 10,015 dermoscopic images across seven lesion classes Actinic Keratoses and Intraepithelial Carcinoma (AKIEC), Basal Cell Carcinoma (BCC), Benign Keratosis-like Lesions (BKL), Dermatofibroma (DF), Melanoma (MEL), Melanocytic Nevi (NV), and Vascular Lesions (VASC). While several studies have explored ensemble learning and XAI techniques independently for skin lesion classification, no existing work has combined heterogeneous ensemble fusion with quantitative trustworthiness validation using TMAI alongside multi-method XAI analysis on the HAM10000 dataset. Furthermore, we assess the proposed methodology on the ISIC 2019 benchmark dataset using a separately trained model to evaluate its generalizability across datasets of varying scale and complexity.

The key contributions of this study are summarized as follows:

- We propose a novel heterogeneous weighted soft voting ensemble model with complementary feature-extraction strategies that integrate compound scaling, residual learning, and dense connectivity.
- A novel class-specific augmentation designed to improve minority class representations, with differentiated transformation parameters for AKIEC, DF, and VASC, which addresses severe class imbalance.
- A progressive dropout regularization scheme (0.65 → 0.55 → 0.45) with batch normalization is incorporated in a custom classification head for all three architectures to prevent overfitting and ensure stable training.
- Class weighted Focal Loss (α = 1, γ = 2.2) applied to focus training on challenging minority samples, attaining perfect classification for BCC (100%, 35/35; 95% CI: 90.1%–100.0%), VASC (100%, 13/13; 95% CI: 77.2%–100.0%), and DF (87.50%, 7/8; 95% CI: 52.9%–97.8%) and 99.77% accuracy for MEL.
- To the best of our knowledge, this is the first study to quantitatively validate ensemble explainability on HAM10000 using TMAI-based trustworthiness evaluation, which objectively measures whether XAI explanations focus on clinically relevant lesion regions. This constitutes the main innovative contribution of the proposed framework, distinguishing it from all existing ensemble-based skin lesion classification methods.

The rest of the paper is organized as follows. Section 2 presents related work on deep learning-based skin lesion classification, transfer learning, and XAI methods. Section 3 presents the proposed methodology, which covers dataset description, preprocessing, augmentation, TL architecture, ensemble fusion, loss function, training protocol, and XAI with TMAI evaluation. Section 4 presents the experimental results including classification performance, confusion matrix analysis, ROC curve analysis, feature space visualization, XAI analysis, ablation study, statistical analysis, cross-validation analysis, and generalizability evaluation on the ISIC 2019 dataset. Section 5 concludes the paper with a summary of findings and future research directions.

## 2 Related work

This section presents existing deep learning approaches for automated skin lesion classification, along with explainable AI techniques and ensemble learning methods, highlighting the limitations that motivate the proposed framework. The work in Groh et al. (9) introduces a physician-machine collaboration framework that uses a DL model to assist dermatologists in diagnosing skin diseases. The primary aim of the research is to identify how AI-assisted decisions can support diagnostic accuracy and bias physician expertise levels and skin tones. The experiment was conducted on a curated dataset of 364 dermoscopic images across 46 skin diseases, annotated with Fitzpatrick skin labels. Experimental findings show that the proposed model achieved over 33% and 69% diagnostic accuracy for specialists and primary-care physicians. However, the work can be extended by integrating XAI techniques and using transfer learning models on different datasets to build more transparent and explainable skin lesion classifiers. In Abohashish et al. (10), the authors proposed a hybrid DL model combining LSTM and CNN layers for automatic classification of melanoma and non-melanoma. LSTM helps capture temporal dependencies from patch-wise sequences, and CNN extracts spatial features. The primary aim of the research is to identify a more robust classification approach that helps overcome the limitations of single-architecture models, especially in handling complex skin lesion patterns. The experiments were carried out on the HAM10000 dataset, and the proposed model achieves an accuracy of 96.21%, precision of 94.75%, recall of 94.93%, AUC of 0.99, and F1-score of 95.55%. However, the authors have not provided interpretable visual explanations of model decisions. Another study in Cheng et al. (11) proposed MobileNet-MFS, which replaces the standard Squeeze-and-Excitation module with a novel fused spatial-channel attention mechanism integrated with a multi-scale feature extraction module for skin cancer image classification. The proposed model captures local receptive-field features and global spatial-channel dependencies in dermoscopic skin lesion images. Experiments were performed on the ISIC-2019 dataset, and the proposed model achieved an accuracy of 87.3%, precision of 0.8732, recall of 0.8751, and an F1-score of 0.8730, outperforming MobileNetV3, DenseNet-121, ResNet34, and ShuffleNetV2. However, the proposed work can enhance model trustworthiness and clinical interpretability. The work in Brinker et al. (12) introduces the first publicly available Melanoma Classification Benchmark, which contains dermoscopic (MClass-D) and non-dermoscopic (MClass-ND) images. The objective of the research is to identify a reliable reference standard for evaluating binary melanoma classification algorithms by benchmarking against the diagnostic performance of 157 German dermatologists. The experimental implementation was carried out on two open-source test sets of 100 dermoscopic and 100 clinical images each, which contain 80 nevi and 20 biopsy-verified melanomas, sourced from the ISIC archive and MED-NODE database. The experimental findings show that the proposed benchmark reveals a sensitivity of 74.1% and an ROC of 0.671 for dermoscopic images, and a sensitivity of 89.4% and an ROC of 0.769 for clinical images. The proposed work can be extended to multi-ethnic skin tone datasets to improve benchmark generalizability. Another research effort (13) proposed a study evaluating Google's Inception V4 CNN architecture against an international group of 58 dermatologists for dermoscopic melanoma classification by using a two-level reader study design. During this process, the authors used a 100-image dermoscopic test set assessed at two levels. Level I used dermoscopy only, whereas Level II used additional clinical information and images to calculate sensitivity, specificity, and ROC-AUC. The proposed model used a dataset sourced from dermoscopic images and was benchmarked against the top five algorithms from the ISBI 2016 challenge. This study achieved a CNN ROC-AUC of 0.82. However, the authors did not provide any information on the explainability of the CNN decision-making process.

The work in Moura et al. (14) proposed a computational methodology that combined Asymmetry, Border, Color, and Diameter with pre-trained CNN features to classify skin lesions as melanoma or non-melanoma using dermoscopic images. During this process, the authors used gain ratios for feature selection and then fed the selected features into a Multi-Layer Perceptron classifier to perform binary skin lesion classification. In this model, the authors formed a new dataset by combining two publicly available databases to validate the proposed methodology. The results discovered in this study achieve an accuracy of 94.9% and a Kappa index of 89.2%. However, the proposed work can be extended to multi-class classification, as the binary melanoma vs. non-melanoma formulation limits practical utility in a clinical environment where several lesion types must be distinguished simultaneously. Another important study in Jaisakthi et al. (15) proposed an automated deep convolutional neural network-based classification system to differentiate melanoma from non-melanoma skin cancer using dermoscopy images. During this process, the authors used the EfficientNet architecture with a Light Gradient Boosting Machine classifier, which was later fine-tuned with contextual metadata fusion. The Ranger optimizer combined with RAdam and Lookahead was used to reduce hyperparameter tuning and improve convergence. The experiments were carried out on the ISIC-2019 and ISIC-2020 datasets, achieving an AUC-ROC score of 0.9681 using EfficientNet-B6 with the Ranger optimizer. The study addresses only binary classification and does not extend to multi-class skin lesion categories. In Kawahara et al. (16), the authors proposed a multi-task, multi-modal deep convolutional neural network. In this work, the authors used InceptionV3 as the base feature extractor. They introduced a multi-modal, multi-task loss function trained across different combinations of dermoscopic images, clinical images, and patient metadata. Since there is a severe class imbalance in the dataset, mini-batches were sampled and weighted by label frequency. The model supports discriminant region localization and multi-modal image retrieval using compact feature vectors. This model uses a dataset containing 1,011 dermoscopic lesion samples; the experimental results report an average diagnostic AUROC of 0.89 with combined multi-modal input and a melanoma classification AUROC of 0.86 via direct diagnosis. However, the authors provide no information on modern explainability, and the dataset is small at just over 1,000 cases, which will limit generalizability to broader clinical deployment. Interestingly, the recent study in Nawaz et al. (17) proposed a novel custom deep convolutional neural network architecture, FCDS-CNN. The authors employed data augmentation techniques such as rotation, flipping, scaling, and zooming, along with inverse-frequency class weighting, to mitigate the dominance of majority classes during training. The proposed FCDS-CNN was trained from scratch with the Adam optimizer, a learning rate of 0.001, a batch size of 32, and trained for 40 epochs. Four pre-trained baseline models, ResNet152V2, EfficientNetV2B0, InceptionResNetV2, and MobileNetV3, were evaluated with the same preprocessing and hyperparameter settings. This model uses the HAM10000 dataset, and the proposed model achieved a precision of 0.96, recall of 0.96, F1-score of 0.97, and AUC of 0.97. The effect of different augmentation strategies on rare lesion classes was not focused on. Another significant study in Halder et al. (18) proposes a fuzzy rank deep ensemble model using Xception, InceptionResNetV2, and MobileNetV2, with fuzzy logic-based rank fusion to address multiple uncertainties and class imbalances in the data. The objective is to develop a robust multi-class skin cancer classifier by combining several transfer learning frameworks within an adaptive fuzzy ensemble strategy. The experiments were carried out on the HAM10000 dataset and DermaMNIST dataset. The findings show the proposed model achieves an accuracy of 95.14% on HAM10000 and 78.25% on DermaMNIST. The author applied only Grad-CAM to interpret model decisions. The proposed work can be extended in the future by using several advanced XAI methods such as LIME and SHAP along with Grad-CAM to provide class-wise explainability. Recent studies explored complementary approaches for automated skin lesion classification. SNC_Net (19) integrates handcrafted and deep learning-based features from dermoscopic images and performs eight-class skin cancer classification on the ISIC 2019 dataset, achieving an accuracy of 97.81% and demonstrating the potential of combining complementary feature-extraction strategies that pair naturally with ensemble-based approaches. Another interesting work in Majid et al. (20) proposes the Modified Whale Optimization Algorithm, integrated with an ensemble of SqueezeNet and InceptionResNet-V2 for multiclass skin cancer classification, achieving accuracies of 95.48% and 98.59% on benchmark datasets, highlighting alternative optimization-based design choices for skin lesion classification. The authors in Ain et al. (21) proposed a lightweight hybrid deep learning framework (DenseNet121 + ConvNeXt-Tiny) with edge enhancement algorithms employing contrast-limited adaptive histogram equalization and bilateral filters for monkeypox lesion detection, achieving 97% accuracy and 99% precision, demonstrating the potential of advanced preprocessing and hybrid deep learning pipelines for skin lesion image analysis beyond dermoscopy. However, XAI methods are not explored to provide interpretable explanations of model predictions.

The reviewed literature shows mainly three critical gaps that hinder the clinical deployment of existing skin lesion classification systems. First, most studies focus on binary classification of melanoma vs. non-melanoma, but in real time doctors need to identify all seven skin disease types. Second, although ensemble and TL approaches have demonstrated better performance, most studies do not validate explainability. In many cases, authors omit XAI entirely, or apply only one type of XAI method without quantitative validation. Third, no study has quantitatively validated the trustworthiness of XAI explanations on the HAM10000 dataset using a proper numeric metric. To address these problems, this study proposes a weighted soft-voting ensemble using EfficientNet-B3, ResNet101, and DenseNet201 for seven-class skin lesion classification, with complementary feature-extraction strategies integrating compound scaling, residual learning, and dense connectivity. Implementation of class-specific augmentation designed to improve minority class representations, with differentiated transformation parameters for AKIEC, DF, and VASC. To the best of our knowledge, this work is the first-ever approach to justify the reliability of TMAI-based validation, resulting in ensemble-based explainability on the HAM10000 dataset. The framework evaluates the explanations generated from three complementary XAI methods: Grad-CAM, LIME, and OSA. Furthermore, the proposed methodology is additionally assessed on the ISIC 2019 benchmark dataset using a separately trained model to evaluate its generalizability across datasets of varying scale and complexity.

## 3 Proposed model and methodology

This section presents the proposed methodology, covering dataset description, preprocessing, augmentation, TL architecture, ensemble fusion, training configuration, and XAI evaluation with the TMAI framework.

### 3.1 Dataset description and characteristics

#### 3.1.1 HAM10000 dataset

**Figure 1:**
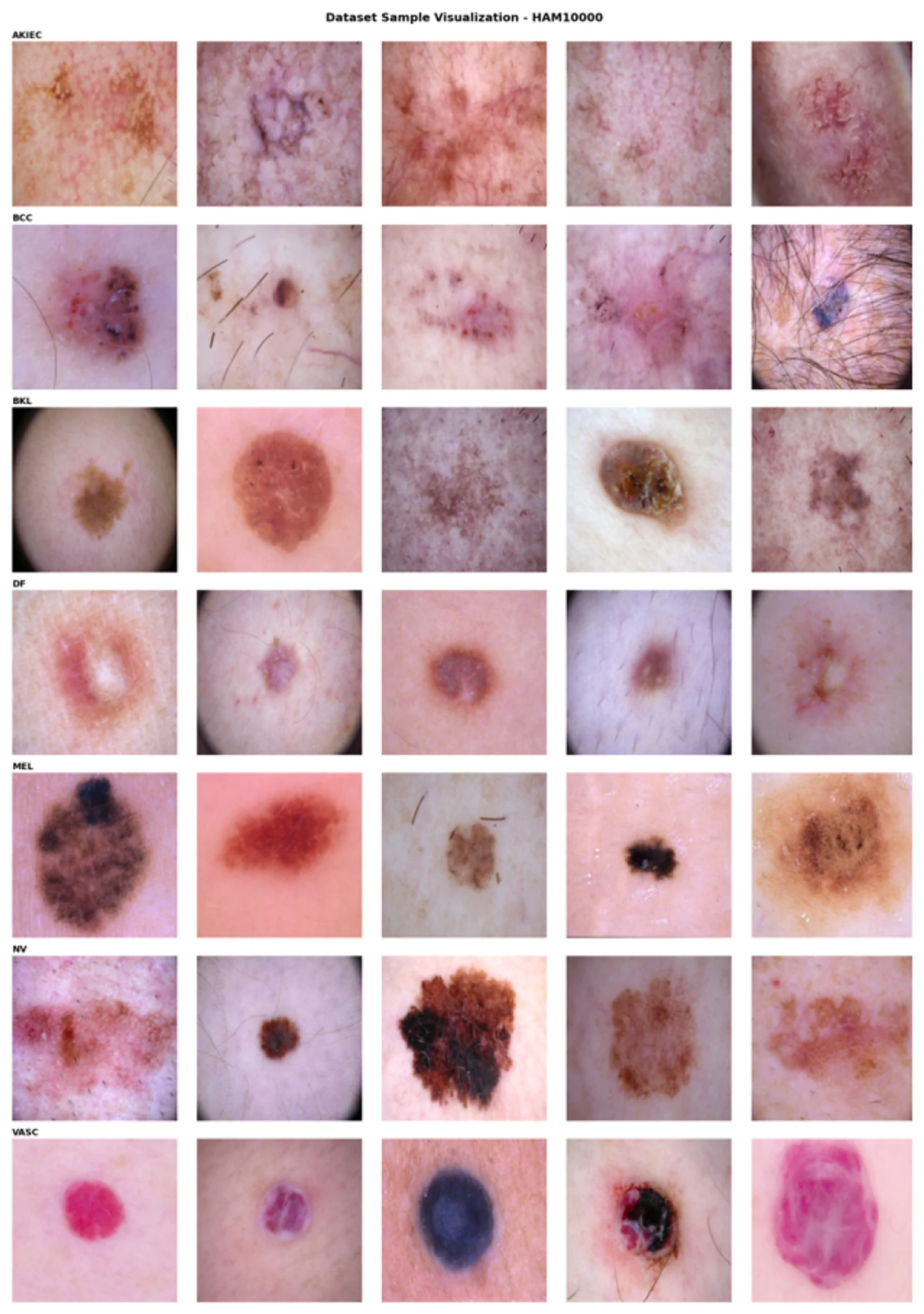
Representative sample images from the HAM10000 dataset across all seven diagnostic categories.

The HAM10000 dataset used in this research is a collection of 10,015 dermatoscopic images of pigmented lesions of seven diagnostic categories (22). The clinically significant skin conditions for the dataset are Actinic keratoses and intraepithelial carcinoma (AKIEC), Basal cell carcinoma (BCC), Benign keratosis-like lesions (BKL), Dermatofibroma (DF), Melanoma (MEL), Melanocytic nevi (NV), and Vascular lesions (VASC). Some of the sample images from each class are given in Figure 1. The dataset shows significant class imbalance reflecting real clinical distribution, as shown in Table 1. NV is the largest class with 6,705 images; DF has only 115 images. MEL, the most clinically important class, contains 1,113 images, followed by BKL with 1,099 images, BCC with 514 images, AKIEC with 327 images, and VASC with 142 images. This imbalance requires special strategies to ensure the models perform better across all classes, especially important minority classes such as AKIEC, DF, and VASC.

**Table 1:** HAM10000 dataset specifications.

Characteristic	Value
Total images	10,015
Unique lesions	7,470
Diagnostic categories	7
Color space	RGB (24-bit)
File format	JPEG
Magnification	10 × –20 × dermoscopy
Verification method	Histopathology, expert consensus, confocal microscopy

#### 3.1.2 ISIC 2019 dataset

We used the ISIC 2019 dataset (23) to assess the generalizability of the proposed ensemble methodology by independently retraining the framework on a different dermoscopic dataset. It contains 25,331 dermoscopic images across eight diagnostic categories: MEL, NV, BCC, AK, BKL, DF, VASC, and squamous cell carcinoma (SCC). All the images were resized to 224 × 224 pixels during preprocessing. We split the dataset into 80% training and 20% validation sets using stratified sampling to maintain class proportions. Because publicly available ISIC 2019 metadata does not provide explicit lesion-level identifiers, we performed the split at the image level using stratified sampling.

### 3.2 Data pre-processing and normalization

All images of the HAM10000 dataset were preprocessed in three sequential steps (resizing, color space conversion, and pixel-wise normalization). Images were resized from their original varying dimensions to a uniform resolution of 224 × 224 pixels using bilinear interpolation, which is shown in Equation 1:


$$\begin{array}{l} I_{\text{resized}}\left(x,y\right)=\overset{1}{\underset{i=0}{\sum }}\overset{1}{\underset{j=0}{\sum }}I_{\text{original}}\left(x_{i},y_{j}\right)\cdot w_{i}\cdot w_{j} \end{array}$$ (1)

where *I*_original_ represents the input image and (*x, y*) are considered as the target pixel coordinates (*x*_*i*_, *y*_*j*_) denotes as four nearest neighboring pixels and *w*_*i*_, *w*_*j*_ are considered as the interpolation weights. This resolution matches the input requirements of ResNet101 and DenseNet201, which were pre-trained on ImageNet. It is acknowledged that EfficientNet-B3 was originally designed for 300 × 300 pixel inputs. However, we resized all images to 224 × 224 pixels in this study to maintain consistent input resolution across all three backbone architectures and reduce computational overhead. This resolution is the standard input size for ResNet101 and DenseNet201, ensuring fair comparison within the ensemble. Evaluation at the native 300 × 300 resolution is identified as a future research direction. All images were converted to RGB color space with 24-bit color depth and stored as floating-point tensors with pixel values ranging from [0, 1].

Pixel-wise normalization was then applied using channel-wise mean and standard deviation values calculated from the HAM10000 training set, which is defined in Equation 2:


$$\begin{array}{l} {Ĩ}_{c}=\frac{I_{c}-\mu_{c}}{\sigma_{c}} \end{array}$$ (2)

where Ĩ_*c*_ is the normalized image for channel *c* ∈ {*R, G, B*}, *I*_*c*_ means the original pixel intensity, μ_*c*_ is taken as channel mean and σ_*c*_ is channel standard deviation. The normalized values for HAM10000 are μ = [0.763, 0.546, 0.570] and σ = [0.141, 0.153, 0.170] for red, green, and blue channels. The high red-channel mean reflects the characteristic pink-to-brown color patterns of dermoscopic images due to erythema and pigmentation. Using dataset-specific normalization instead of standard ImageNet statistics improves model performance because it better matches the data distribution to the dermoscopy domain.

### 3.3 Dataset partitioning and stratified sampling

To ensure a balanced distribution of classes in both the training and validation sets, we used stratified sampling. This method ensures that the number of samples collected from each diagnostic category in both splits is close to that for which the original dataset (samples) is distributed to avoid bias from unbalanced sets. Formally, for each class *c* ∈ {AKIEC, BCC, BKL, DF, MEL, NV, VASC}, the class proportion is maintained across splits as shown in Equation 3:


$$\begin{array}{l} p_{c}^{\text{train}}\approx p_{c}^{\text{val}}\approx p_{c} \end{array}$$ (3)

where $p_{c}=\frac{n_{c}}{N}$ is considered as proportion of class *c* in full dataset, $p_{c}^{\text{train}}$ and $p_{c}^{\text{val}}$ represents corresponding proportions in training set and validation set, *n*_*c*_ denotes number of samples for class *c*, and *N* is considered as total dataset size. This strategy ensures that minority classes such as DF and VASC are appropriately represented in the validation set despite their limited sample counts, leading to reliable performance evaluation across all classes. It also prevents the validation set from being dominated by NV, which can increase overall accuracy while hiding poor performance on important minority classes. The partitioning was done at the lesion level, ensuring that all images of a particular lesion are present in the training or validation set, which helps prevent data leakage. This resulted in 8,012 training images from 5,976 unique lesions and 2,003 validation images from 1,494 unique lesions. The experimental evaluation was performed using the unduplicated validation subset generated after lesion-level preprocessing. Specifically, we first identified lesions represented by a single dermoscopic image, then created the validation set by performing a stratified train-validation split on this unduplicated dataset. Consequently, we computed the confusion matrix and all reported performance metrics on this 1,103-image validation subset to ensure unbiased per-class performance evaluation.

### 3.4 Data augmentation and class balancing

We applied data augmentation to the training set to increase data diversity and enhance model generalization. This process incorporated spatial transformations, photometric adjustments, and advanced regularization methods. Spatial transformations included random horizontal flip (*p* = 0.5), random vertical flip (*p* = 0.3), random rotation (±15°), random affine transformation with translation (±10%) and scale variation (0.9–1.1). These transformations replicate patient orientations, camera angles, and image capture distances typically encountered in real-world clinical environments. The spatial transformation pipeline is given as follows in Equation 4:


$$\begin{array}{l} I_{\text{spatial}}=T_{\text{affine}}○T_{\text{rotate}}○T_{\text{vflip}}○T_{\text{hflip}}\left(I_{\text{original}}\right) \end{array}$$ (4)

where ○ is considered as function composition and each transformation *T* is randomly applied according to its specified probability. Photometric augmentation involved color jitter with brightness (±0.2), contrast (±0.2), saturation (±0.2), and hue (±0.1) to simulate lighting and imaging variations of different dermatoscope models. We added random erasing (*p* = 0.2) to improve robustness to dermoscopic artifacts such as hair, air bubbles, and ruler markings. Mixup augmentation was applied to 30% of training batches, where new samples were created as convex combinations of image pairs.

To handle severe class imbalance, we applied a class-specific augmentation strategy to the minority classes AKIEC, DF, and VASC using more aggressive transformation parameters than for the standard classes, as detailed in Table 2. This approach increases minority class sample diversity without simple duplication, helping the model learn more robust features for these minority classes. No augmentation was applied for the validation set, ensuring unbiased performance evaluation.

**Table 2:** Class-specific augmentation parameters.

Transformation	Standard classes	Minority classes
	(BCC, BKL, MEL, and NV)	(AKIEC, DF, and VASC)
Horizontal flip	*p* = 0.5	*p* = 0.7
Vertical flip	*p* = 0.3	*p* = 0.5
Rotation	±15°	±20°
ColorJitter (Brightness)	±0.2	±0.3
ColorJitter (Contrast)	±0.2	±0.3
ColorJitter (Saturation)	±0.2	±0.3
ColorJitter (Hue)	±0.1	±0.15
Affine translation	±0.1	±0.15
Affine scale	(0.9, 1.1)	(0.85, 1.15)
Random erasing	*p* = 0.2	*p* = 0.3

### 3.5 Transfer learning architecture

This study uses an ensemble of three CNN models: EfficientNet-B3, ResNet101, and DenseNet201, pre-trained on ImageNet-1K and fine-tuned for seven-class skin lesion classification. We chose these models for their complementary design philosophies: compound scaling for efficiency, residual learning for depth, and dense connectivity for feature reuse. Each model uses a custom classification head, with all layers fine-tuned end-to-end to adapt from natural images to dermoscopic images. Although ImageNet consists of natural images instead of medical images, pre-trained models learned from ImageNet have been used in many studies for dermoscopic image classification through transfer learning. The low-level features learned from ImageNet, such as edges, textures, and color patterns, can be effectively applied to different domains including medical imaging. In dermoscopy images, these features matter because skin lesions often show unique texture patterns, color differences, and irregular borders that help with diagnosis. Furthermore, fine-tuning on the HAM10000 dataset adapts the higher-level feature representations to dermoscopy-specific patterns, bridging the domain gap between natural and medical images. This approach is especially useful when medical datasets are limited in size, as ImageNet pre-training provides a strong initialization for learning and helps in reducing the risk of overfitting, leading to better performance than training a model from scratch. Task-specific pre-training on larger dermoscopic datasets improves the proposed framework's generalization, as shown by its strong performance on the independent ISIC 2019 dataset.

#### 3.5.1 Architecture selection and specifications

**EfficientNet-B3** applies compound scaling to balance network depth, width, and resolution. It produces 1,536-dimensional feature representations through mobile inverted bottleneck convolution (MBConv) blocks with squeeze-and-excitation attention mechanisms, allowing better channel-wise feature adjustment to capture fine color and texture details, which is important in dermoscopy. The feature extraction process is defined in Equation 5:


$$\begin{array}{l} F_{\text{EfficientNet}}=\text{GAP}({\text{MBConv}}_{7}({\text{MBConv}}_{6}\\ \;(\cdots {\text{MBConv}}_{1}({\text{Conv}}_{\text{stem}}(I)))))\in {\mathbb{R}}^{1536} \end{array}$$ (5)

where Conv_stem_ is the initial 3 × 3 convolution with stride 2, MBConv_*i*_ denotes the *i*-th stage, and GAP performs global average pooling to produce the 1,536-dimensional feature vector. The model was instantiated using the timm library (efficientnet_b3) with ImageNet pre-trained weights. The detailed feature extraction procedure is presented in Algorithm 1.

Algorithm 1 EfficientNet-B3 feature extraction (29) 
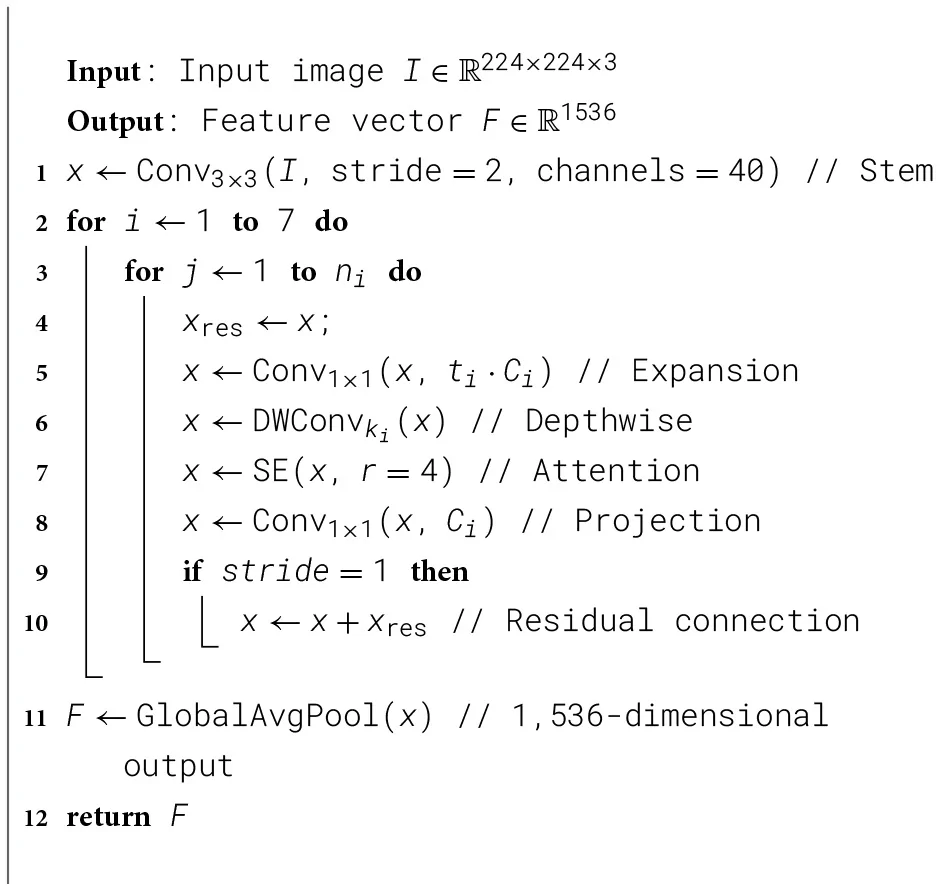

**ResNet101** is a 101-layer deep residual network that uses identity skip connections to allow gradient flow through very deep networks. It generates 2,048-dimensional feature vectors through four residual stages containing 3, 4, 23, and 3 bottleneck blocks. The bottleneck blocks implement the residual mapping given in Equation 6:


$$\begin{array}{l} H\left(x\right)=F\left(x,\left\{W_{i}\right\}\right)+x \end{array}$$ (6)

where $F\left(x,\left\{W_{i}\right\}\right)$ denotes the series of 1 × 1, 3 × 3, and 1 × 1 convolutions, and *x* is the identity mapping. The backbone processes 224 × 224 × 3 inputs through progressive spatial downsampling (56 × 56 → 28 × 28 → 14 × 14 → 7 × 7), increasing the number of channel dimensions (64 → 256 → 512 → 1, 024 → 2, 048). The model is implemented using torchvision.models.resnet101 with ImageNet pre-trained weights. The feature extraction process follows Algorithm 2.

Algorithm 2 ResNet101 feature extraction (30). 
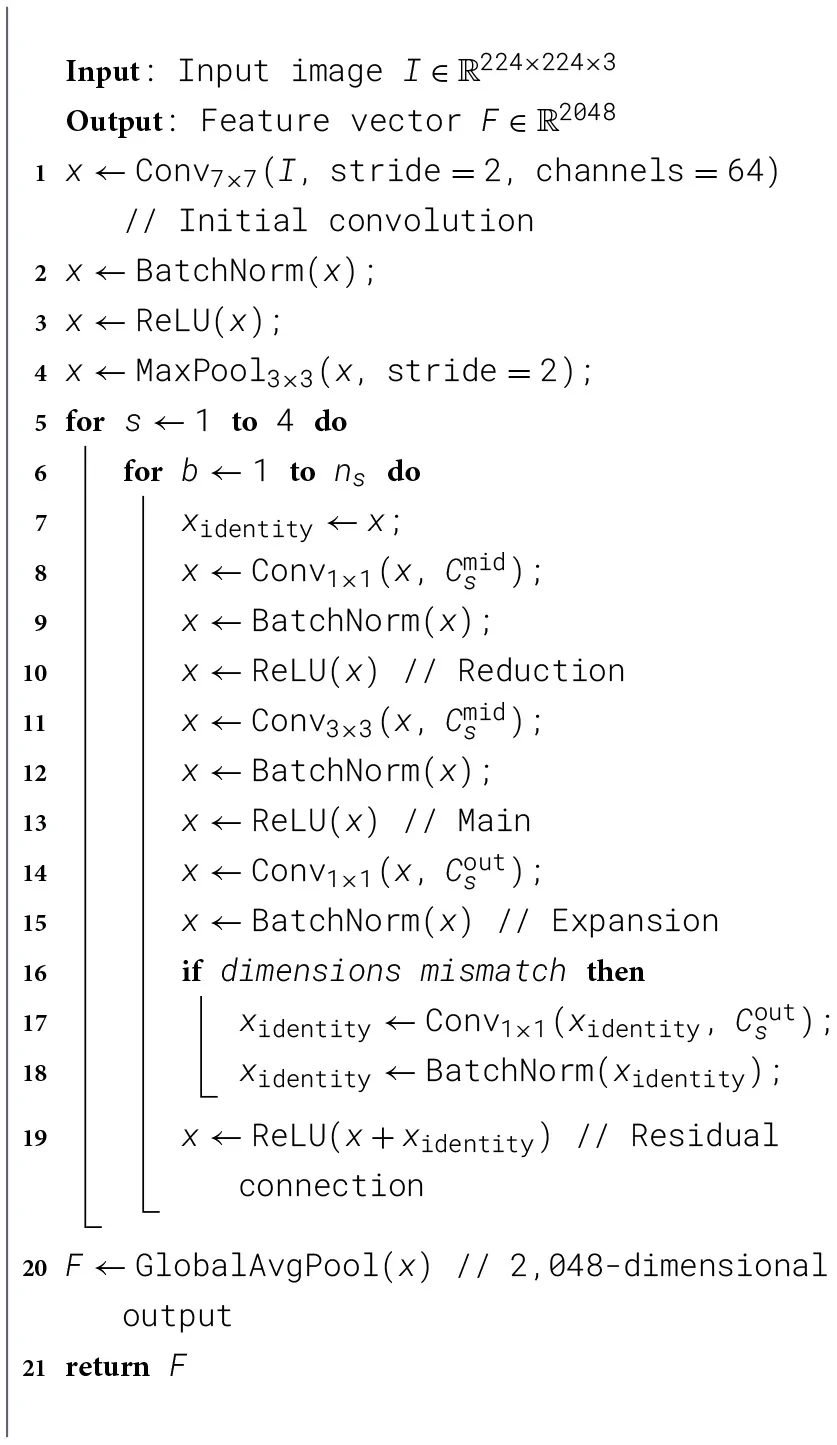

Stage parameters: Stage 1: *n*_1_ = 3, $C_{1}^{\text{mid}}=64$, $C_{1}^{\text{out}}=256$; Stage 2: *n*_2_ = 4, $C_{2}^{\text{mid}}=128$, $C_{2}^{\text{out}}=512$; Stage 3: *n*_3_ = 23, $C_{3}^{\text{mid}}=256$, $C_{3}^{\text{out}}=1024$; Stage 4: *n*_4_ = 3, $C_{4}^{\text{mid}}=512$, $C_{4}^{\text{out}}=2048$.

**DenseNet201** uses dense connections where each layer receives feature maps from all previous layers and produces 1920-dimensional feature vectors. This reuse helps retain fine-grained spatial details, which are essential for distinguishing similar lesion types. The architecture has four dense blocks with 6, 12, 48, and 32 layers, separated by transition layers with 0.5 compression. The model is implemented using torchvision.models.densenet201 with ImageNet pre-trained weights. The feature extraction is outlined in Algorithm 3.

Algorithm 3 DenseNet201 feature extraction (31). 
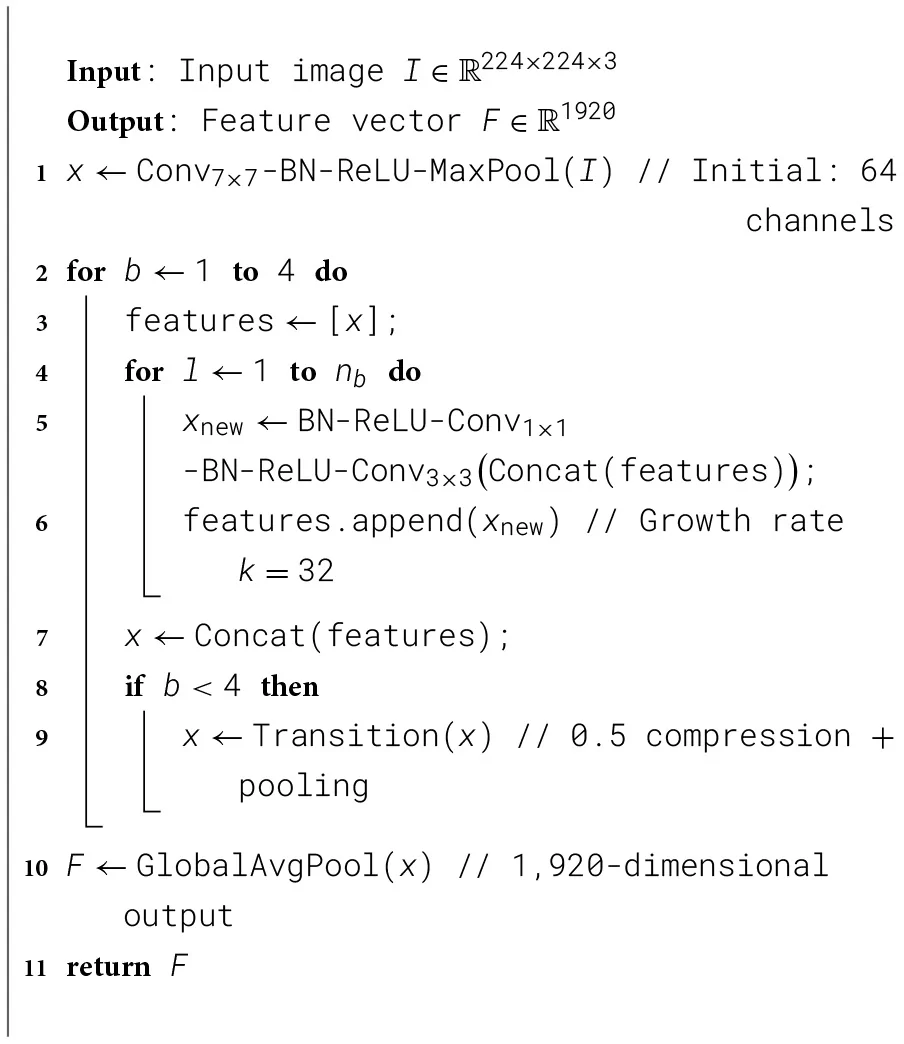

*n*_1_ = 6, *n*_2_ = 12, *n*_3_ = 48, *n*_4_ = 32 layers; growth rate *k* = 32.

All three models accept 224 × 224 × 3 input images normalized using dataset-specific statistics (μ = [0.763, 0.546, 0.570], σ = [0.141, 0.153, 0.170]).

#### 3.5.2 Custom classification head

The ImageNet classification layers of all three models are replaced with the same custom classification head for the seven-class skin lesion classification, resulting in a consistent final decision-making process. The unified architecture is defined in Equation 7:


$${ℱ}_{\text{classifier}}:{\mathbb{R}}^{d}\overset{\text{Layer 1}}{\to }{\mathbb{R}}^{512}\overset{\text{Layer 2}}{\to }{\mathbb{R}}^{256}\overset{\text{Layer 3}}{\to }{\mathbb{R}}^{7}$$ (7)

where *d* ∈ {1, 536, 1, 920, 2, 048} is the specific backbone feature dimension for EfficientNet-B3, DenseNet201, and ResNet101, and the output dimension 7 corresponds to the seven diagnostic classifications. The layer specifications are given in Table 3. A progressive dropout strategy (0.65 → 0.55 → 0.45) provides stronger regularization in the initial layers with the highest feature dimensionality and is slowly reduced in deeper layers to preserve important information. Batch normalization is applied after each linear layer to stabilize training as defined in Equation 8:


$$\begin{array}{l} {\hat{x}}_{i}=\frac{x_{i}-\mu_{B}}{\sqrt{\sigma_{B}^{2}+\epsilon }}\cdot \gamma +\beta \end{array}$$ (8)

where $\mu_{B}$ and $\sigma_{B}^{2}$ are the mini-batch mean and variance, ϵ = 10^−5^ is considered as numerical stability constant, and γ, β denotes learnable parameters. ReLU activation ReLU(*x*) = max(0, *x*) is applied after the batch normalization layer.

**Table 3:** Classification head layer specifications.

Layer	Operation	Input dim	Output dim
Layer 1	Dropout (*p* = 0.65)	*d*	*d*
	Linear	*d*	512
	BatchNorm1d	512	512
	ReLU	512	512
Layer 2	Dropout (*p* = 0.55)	512	512
	Linear	512	256
	BatchNorm1d	256	256
	ReLU	256	256
Output	Dropout (*p* = 0.45)	256	256
	Linear	256	7

#### 3.5.3 Fine-tuning configuration

All three models were fine-tuned, allowing gradient updates through all layers, and each uses a custom classification head, fine-tuned end-to-end to adapt from natural images to dermoscopic images. This allows the network to adapt learned features at all levels; lower layers adopt edge and texture recognition to dermatoscopic features such as pigment networks and vascular structures, and higher layers learn new task-related features to classify the seven diagnostic categories. Full fine-tuning is crucial for dermoscopy because it has distinctive characteristics, including special illumination, magnification effects, and important color patterns like blue-white veil and regression structures. Model-specific learning rates are used as shown in Table 4: 1.0 × 10^−4^ for EfficientNet-B3, 7.5 × 10^−5^ for DenseNet201, and 5.0 × 10^−5^ for ResNet101, selected through initial validation to balance learning speed and training stability.

**Table 4:** Model-specific learning rates for fine-tuning.

Model	Learning rate
EfficientNet-B3	1.0 × 10^−4^
DenseNet201	7.5 × 10^−5^
ResNet101	5.0 × 10^−5^

### 3.6 Weighted ensemble fusion

A weighted soft voting ensemble was implemented to combine predictions from three CNN backbone models, where each of the CNN models contributes based on its validation performance. For each model *m* ∈ EfficientNet-B3, ResNet101, DenseNet201, the forward pass produces seven-class logit vectors, which are given in Equation 9:


$$\begin{array}{l} f_{m}\left(x\right)={\text{Classifier}}_{m}\left({\text{Backbone}}_{m}\left(x\right)\right)\in {\mathbb{R}}^{7} \end{array}$$ (9)

where Backbone_*m*_ extracts features and Classifier_*m*_ is denoted as custom classification head. The Softmax normalization converts logits into a probability distribution, which is defined in Equation 10:


$$\begin{array}{l} P_{m}\left(c|x\right)=\frac{\exp\left(f_{m}{\left(x\right)}_{c}\right)}{\sum_{j=1}^{7}\exp\left(f_{m}{\left(x\right)}_{j}\right)} \end{array}$$ (10)

where *f*_*m*_(*x*)_*c*_ is considered as the logit for class *c* from model *m*, ensuring $\overset{7}{\underset{c=1}{\sum }}P_{m}\left(c|x\right)=1$. The final ensemble prediction is computed as a weighted combination of all these probability distributions shown in Equation 11:


$$\begin{array}{l} P_{\text{ensemble}}\left(c|x\right)=\overset{M}{\underset{m=1}{\sum }}w_{m}\cdot P_{m}\left(c|x\right) \end{array}$$ (11)

where *w*_*m*_ is denoted as weight for model *m*, *M* = 3, and $\overset{M}{\underset{m=1}{\sum }}w_{m}=1$. For the three-model ensemble, this further expands to Equation 12:


$$\begin{array}{l} P_{\text{ensemble}}\left(c|x\right)=w_{\text{E}}\cdot P_{\text{E}}\left(c|x\right)+w_{\text{R}}\cdot P_{\text{R}}\left(c|x\right)+w_{\text{D}}\cdot P_{\text{D}}\left(c|x\right) \end{array}$$ (12)

where *w*_E_ = 0.40, *w*_R_ = 0.20, and *w*_D_ = 0.40 are the empirically determined weights for EfficientNet-B3, ResNet101, and DenseNet201 respectively. The final class prediction is obtained as shown in Equation 13:


$$\begin{array}{l} ŷ=\arg\underset{c\in \left\{1,...,7\right\}}{\max}P_{\text{ensemble}}\left(c|x\right) \end{array}$$ (13)

**Figure 2:**
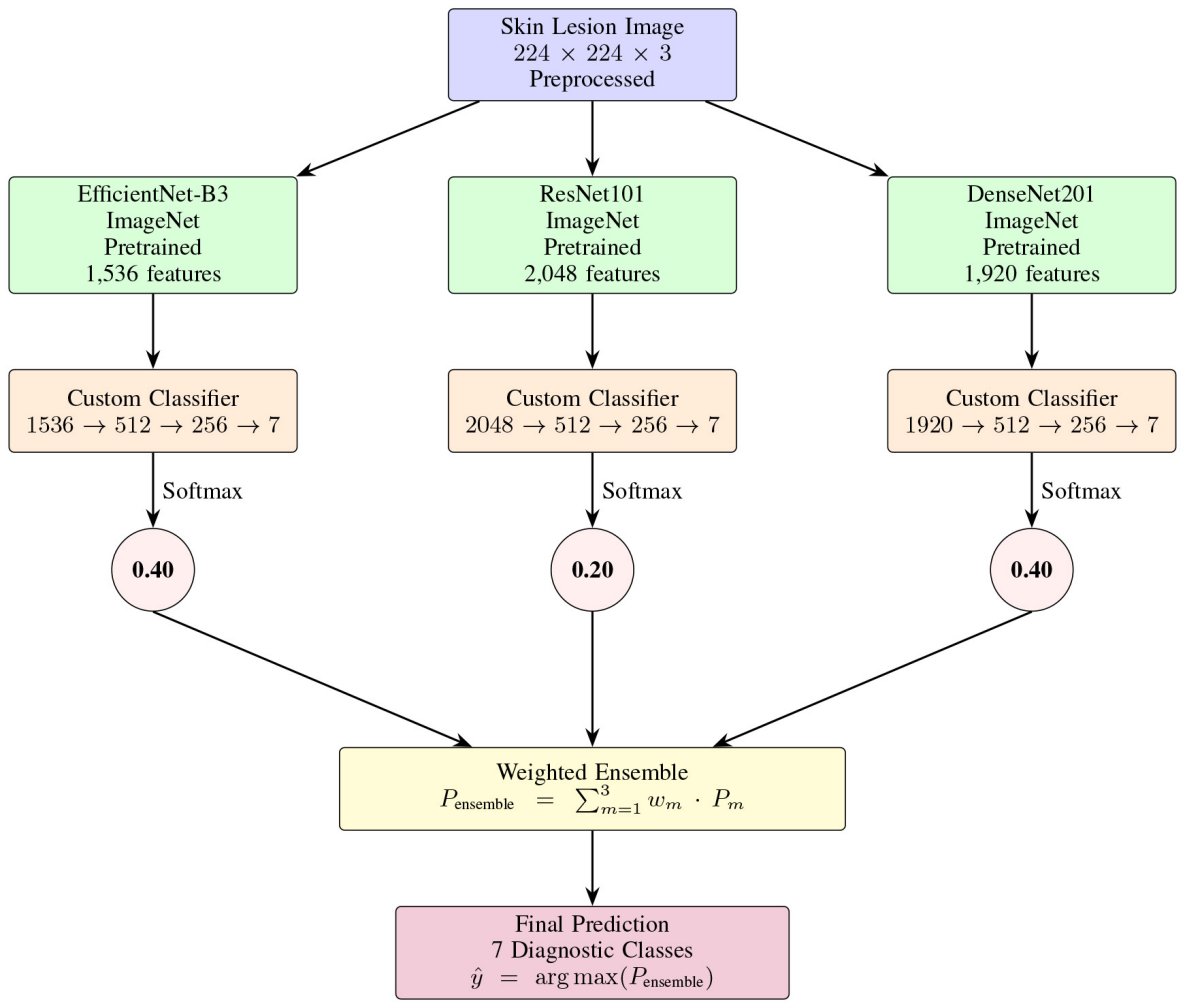
Weighted ensemble architecture combining three CNN backbones (EfficientNet-B3, ResNet101, and DenseNet201) with custom classification heads.

EfficientNet-B3 and DenseNet201 were each assigned a weight of 0.40 based on their better validation performance; ResNet101 was assigned a weight of 0.20 as a supporting model. The final weight configuration is presented in Table 5. The complete ensemble prediction process is described in Algorithm 4. The overall ensemble architecture is depicted in Figure 2. The diagram shows a parallel processing pipeline in which a preprocessed skin lesion image is passed through three pretrained CNN backbone models, each extracting high-dimensional features (EfficientNet-B3: 1,536; ResNet101: 2,048; DenseNet201: 1,920 dimensions). After applying softmax normalization, the output probability distributions are weighted as (0.40, 0.40, and 0.20) and aggregated through weighted summation to provide the final seven-class prediction.

**Table 5:** Ensemble Model Weights.

Model	Weight (*w*_*m*_)	Feature dimension
EfficientNet-B3	0.40	1536
DenseNet201	0.40	1920
ResNet101	0.20	2048
Total	1.00	-

Algorithm 4 Weighted ensemble prediction. 
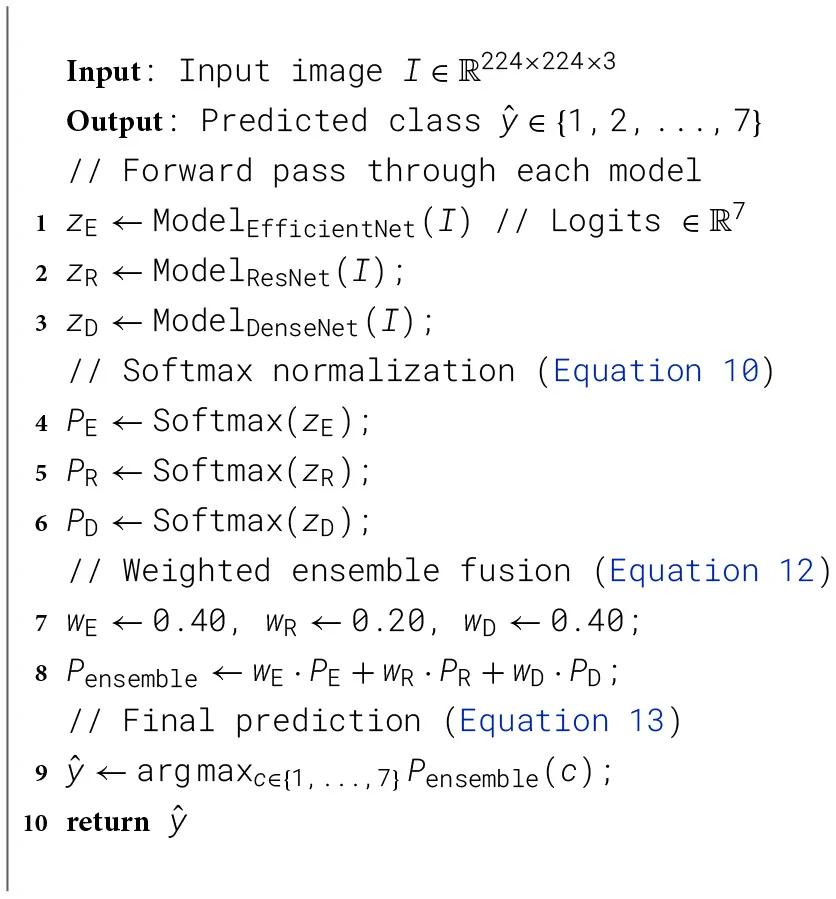

### 3.7 Loss function, optimization and training protocol

#### 3.7.1 Focal loss for class imbalance

The HAM10000 dataset has a high level of class imbalance: NV contains 66.97% of all training samples, whereas the minority classes DF and VASC have only 1.15% and 1.42%, respectively. Standard cross-entropy loss will treat all samples equally, which will lead to majority class bias and poor performance on minority classes. To address these types of issues, Focal Loss (24) was used through two mechanisms: (1) down-weighting easy examples to focus learning on hard misclassified cases, (2) applying class-balancing weights to compensate for frequency imbalance. For a sample with true class *y* and predicted probability *p*_*y*_, the Focal Loss is defined as shown in Equation 14:


$$\begin{array}{l} L_{\text{FL}}\left(p_{y}\right)=-\alpha_{y}{\left(1-p_{y}\right)}^{\gamma }\log\left(p_{y}\right) \end{array}$$ (14)

where α_*y*_ is considered as class weight and γ≥0 controls down weighting of all the easy examples. For seven-class classification the multiclass extension is shown in Equation 15:


$$\begin{array}{l} L_{\text{FL}}=-\frac{1}{N}\overset{N}{\underset{i=1}{\sum }}\alpha_{y_{i}}{\left(1-p_{y_{i}}\right)}^{\gamma }\log\left(p_{y_{i}}\right) \end{array}$$ (15)

where *N* represents the batch size, *y*_*i*_ is called as the true class for sample *i*, and *p*_*y*_*i*__ is denoted as the predicted probability for true class. Hyperparameters were set to α = 1.0 and γ = 2.2, where γ = 2.2 provides a stronger focus on hard examples when compared with the standard γ = 2.0, which was found necessary for the challenging melanoma detection task. The class-specific weights were calculated using inverse frequency with manual tuning as defined in Equation 16:


$$\begin{array}{l} w_{c}=\text{clamp}\left({\left(\frac{N_{\text{total}}}{K\cdot N_{c}}\right)}^{\beta }\cdot \tau_{c},w_{\min},w_{\max}\right) \end{array}$$ (16)

where *N*_*c*_ is considered as sample count for class *c*, *K* = 7, β = 1.0, and weights are constrained to the interval [1.0, 20.0] for ensuring training stability. Additional manually tuned factors τ_*c*_ were applied to provide emphasis on critical minority classes: AKIEC (τ = 7.5), NV (τ = 4.2), VASC (τ = 2.5), BKL (τ = 1.4), DF (τ = 1.1), with MEL and BCC at τ = 1.0. The class-specific multipliers τ_*c*_ were determined based on the class distribution characteristics of the HAM10000 dataset. Classes with severe underrepresentation received higher multipliers to compensate for their limited sample counts. Specifically, AKIEC received the highest multiplier (τ = 7.5) due to its severe underrepresentation, VASC (τ = 2.5), BKL (τ = 1.4), and DF (τ = 1.1) were assigned proportionally based on their degree of underrepresentation, while MEL and BCC retained the default multiplier (τ = 1.0) given their relatively balanced representation. It is important to note that the multiplier τ_NV_ = 4.2 does not constitute up-weighting of the majority class in the conventional sense. The inverse-frequency base weight for NV computes to $\frac{10,015}{7\times 6,705}=0.213$, which is immediately floored to 1.0 by the clamp operation [1.0, 20.0]. Simultaneously, Focal Loss with γ = 2.2 significantly suppresses easy NV samples during training due to their high confidence. The combined effect of the clamp floor and Focal Loss suppression reduces the effective NV learning signal, degrading the model's ability to distinguish NV from visually similar classes, particularly MEL. The τ_NV_ = 4.2 multiplier compensates for this compound suppression effect to maintain sufficient NV feature learning and reduce the clinically dangerous NV-MEL confusion boundary. The final NV weight (4.2) remains substantially lower than AKIEC (7.5), preserving the intended minority-class emphasis throughout training. The weight clamp range of [1.0, 20.0] was applied to prevent extreme weight values that could destabilize the training process.

#### 3.7.2 AdamW optimization and learning rate scheduling

We used the AdamW optimizer (25) with decoupled weight decay to improve generalization. The parameter update rule is defined in Equation 17:


$$\begin{array}{l} m_{t}=\beta_{1}m_{t-1}+\left(1-\beta_{1}\right)g_{t}\\ v_{t}=\beta_{2}v_{t-1}+\left(1-\beta_{2}\right)g_{t}^{2}\\ {\hat{m}}_{t}=\frac{m_{t}}{1-\beta_{1}^{t}},\;{\hat{v}}_{t}=\frac{v_{t}}{1-\beta_{2}^{t}}\\ \theta_{t}=\theta_{t-1}-\eta_{t}\left(\frac{{\hat{m}}_{t}}{\sqrt{{\hat{v}}_{t}}+\epsilon }+\text{λ}\theta_{t-1}\right) \end{array}$$ (17)

where β_1_ = 0.9, β_2_ = 0.999, ϵ = 10^−8^, and weight decay λ = 1.5 × 10^−3^. Model-specific learning rates used: EfficientNet-B3 (η = 1.0 × 10^−4^), DenseNet201 (η = 7.5 × 10^−5^), and ResNet101 (η = 5.0 × 10^−5^). A StepLR scheduler reduced the learning rate by factor of γ = 0.7 every *s* = 10 epochs which is as shown in Equation 18:


$$\begin{array}{l} \eta_{t}=\eta_{0}\cdot \gamma^{\left\lfloor \frac{t}{s}\right\rfloor } \end{array}$$ (18)

This 30% reduction every 10 epochs ensures a balance between early exploration and late-stage fine-tuning. Gradient clipping was applied with threshold θ_max_ = 0.5 to prevent gradient explosions which is defined in Equation 19:


$$g\leftarrow \{\begin{array}{ll} g & \text{if }\|g{\|}_{2}\leq \theta_{\max}\\ \frac{\theta_{\max}}{\|g{\|}_{2}}\cdot g & \text{if }\|g{\|}_{2}>\theta_{\max} \end{array}$$ (19)

Automatic mixed precision training with PyTorch GradScaler used FP16 for computation and FP32 for critical operations, minimizing memory usage and speeding up training by maintaining numerical stability.

#### 3.7.3 Training protocol

All three models were trained simultaneously for up to 50 epochs with a batch size of 32 on an Nvidia GPU with CUDA acceleration; each model maintained its independent optimizer states and learning rate schedule. Mixup augmentation (*p* = 0.3, α = 0.2) was used during training to improve generalization, as shown in Equation 20.


$$\begin{array}{l} \tilde{x}=\text{λ}x_{i}+\left(1-\text{λ}\right)x_{j},\;ỹ=\text{λ}y_{i}+\left(1-\text{λ}\right)y_{j} \end{array}$$ (20)

where λ~Beta(α, α) with α = 0.2. Validation was performed at the end of each epoch using weighted ensemble fusion as defined in Equation 21:


$$\begin{array}{l} P_{\text{ensemble}}\left(y|x\right)=0.40\cdot P_{\text{EfficientNet}}\left(y|x\right)+0.40\cdot P_{\text{DenseNet}}\left(y|x\right)\\ \text{ s }+0.20\cdot P_{\text{ResNet}}\left(y|x\right) \end{array}$$ (21)

Early stopping was utilized with a patience of 12 epochs, minimum improvement threshold δ = 0.0003, and minimum training time duration of 25 epochs. The model checkpoint that had the maximum validation accuracy was saved for the final evaluation.

### 3.8 Explainable AI and trustworthiness evaluation

To increase model interpretability and support clinical trust, we applied three complementary XAI methods to visualize the ensemble model's predictions and validate quantitative trustworthiness using TMAI.

#### 3.8.1 Occlusion sensitivity analysis

Occlusion Sensitivity Analysis measures the importance of various regions in an image by systematically covering all the patches and observing the drop in prediction confidence (26). For each of the positions (*x, y*), a square region of *p*×*p* pixels is replaced with a uniform gray patch (intensity 128), and the sensitivity is calculated as shown in Equation 22:


$$\begin{array}{l} S\left(x,y\right)=P_{\text{ensemble}}\left(c|I\right)-P_{\text{ensemble}}\left(c|{Ĩ}_{x,y}\right) \end{array}$$ (22)

where *I* represents the original image, *c* is considered as predicted class, and Ĩ_*x, y*_ denotes as occluded image. Higher sensitivity values highlight diagnostically important regions. The method uses a patch size of *p* = 28 pixels with a stride of *s* = 14 pixels (50% overlap), which balances computational efficiency with spatial resolution across the 224 × 224 input images.

#### 3.8.2 Gradient-weighted class activation mapping

Grad-CAM produces class specific localization maps by using gradient information from final convolutional layer (27). The importance weight $\alpha_{k}^{c}$ for each feature map *k* is calculated using global average pooling of gradients which is defined by using Equation 23:


$$\begin{array}{l} \alpha_{k}^{c}=\frac{1}{Z}\underset{i}{\sum }\underset{j}{\sum }\frac{\partial y^{c}}{\partial A_{ij}^{k}} \end{array}$$ (23)

where *Z* denotes number of pixels in feature map and $A_{ij}^{k}$ is considered as activation at spatial location (*i, j*) in feature map *k*. The class activation map is shown in Equation 24:


$$\begin{array}{l} L_{\text{GradCAM}}^{c}=\text{ReLU}\left(\underset{k}{\sum }\alpha_{k}^{c}A^{k}\right) \end{array}$$ (24)

The ReLU operation keeps only the features that contribute positively to the target class. In the present study, Grad-CAM was applied to the ensemble model $M_{\text{ensemble}}$ using the final ensemble prediction ĉ as the target class. The class-specific activation maps are generated with respect to the weighted ensemble output, with gradients flowing through the constituent backbone layers (final convolutional head of EfficientNet-B3, denseblock4 of DenseNet201, and layer4 of ResNet101), ensuring that the fused explanation is consistent with the final weighted ensemble prediction of the proposed framework.

#### 3.8.3 Local interpretable model-agnostic explanations

LIME provides model agnostic explanations by learning local linear approximation of the model's behavior for given prediction (28). The input image is divided into *N*_*s*_ = 50 superpixels using SLIC with compactness κ = 10 and smoothing σ = 1. A total of *N*_*p*_ = 2, 000 perturbed samples are generated by randomly switching superpixel visibility, and LIME learns a sparse linear model *g* by constantly minimizing objective which is defined in Equation 25:


$$\begin{array}{l} \xi \left(x\right)=\arg\underset{g\in G}{\min}L\left(f,g,\pi_{x}\right)+\text{Ω}\left(g\right) \end{array}$$ (25)

where L denotes locality-aware loss weighted by proximity kernel π_*x*_, and Ω(*g*) promotes sparsity. The top *K* = 20 influential superpixels are highlighted using minimum weight threshold *w*_min_ = 0.01 to filter out the negligible contributions.

#### 3.8.4 Trustworthiness metric for AI

TMAI quantitatively measures whether the explanations provided by XAI are correctly focused on lesion regions and not background. Automated lesion segmentation uses color based thresholding in the HSV space (*H* ∈ [0, 20], *S* ∈ [10, 150], *V* ∈ [60, 255]) and Otsu's thresholding in LAB space which are combined using bitwise OR and optimized using morphological operations (elliptical kernel 5 × 5). The TMAI score is calculated according to the following Equation 26:


$$\begin{array}{l} \text{TMAI}\left(E,M_{L}\right)={Ē}_{\text{lesion}}-{Ē}_{\text{background}} \end{array}$$ (26)

where Ē_lesion_ and Ē_background_ are the mean normalized explanation values in lesion and background regions (Equation 27, 28):


$$\begin{array}{l} {Ē}_{\text{lesion}}=\frac{1}{\left|L\right|}\underset{\left(i,j\right)\in L}{\sum }E_{\text{norm}}\left(i,j\right),\;L=\left\{\left(i,j\right):M_{L}\left(i,j\right)=1\right\} \end{array}$$ (27)

$$\begin{array}{l} {Ē}_{\text{background}}=\frac{1}{\left|B\right|}\underset{\left(i,j\right)\in B}{\sum }E_{\text{norm}}\left(i,j\right),\;B=\left\{\left(i,j\right):M_{L}\left(i,j\right)=0\right\} \end{array}$$ (28)

where $E_{\text{norm}}=\frac{E-E_{\min}}{E_{\max}-E_{\min}}$. Positive values of TMAI evidence that the explanations are lesion oriented and negative values evidence that the explanations are background oriented. Evaluation was performed on stratified sample of up to 10 images per class using all three XAI methods with all available images used for classes with fewer than 10 validation samples and provides a quantitative comparison of explaining reliability among the seven diagnostic categories.

### 3.9 System architecture and workflow

**Figure 3:**
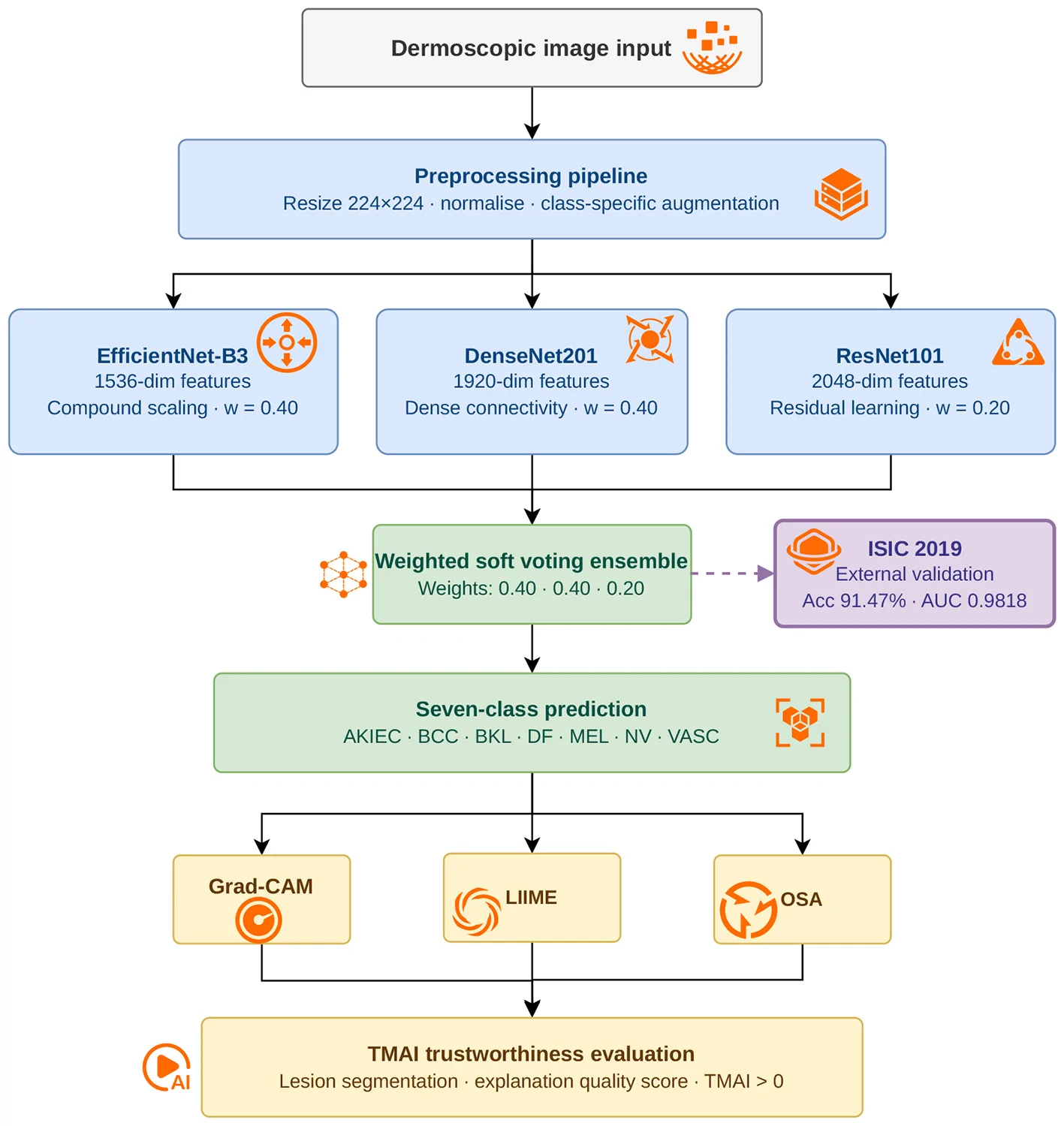
Architecture of the proposed ensemble-based skin lesion classification framework with weighted soft voting fusion and multi-method XAI explainability validation using TMAI.

Figure 3 shows the overall structure of the proposed ensemble-based system of automated skin lesion classification with explainable AI integration. The system contains six stages that convert raw dermoscopic images into trustworthy diagnostic predictions: dataset preparation, preprocessing and normalization, data augmentation, parallel feature extraction using three CNN backbone models, weighted ensemble fusion, and explainability analysis with trustworthiness validation.

#### 3.9.1 End-to-end pipeline

The pipeline starts with the HAM10000 dataset that has 10,015 images across seven classes AKIEC, BCC, BKL, DF, MEL, NV, and VASC. All the images are resized to 224 × 224 pixels and then they are normalized with dataset specific statistics (μ = [0.763, 0.546, 0.570], σ = [0.141, 0.153, 0.170]). During training, augmentation applies geometric transformations (rotation ±15°, horizontal flip *p* = 0.5, vertical flip with *p* = 0.3, affine transforms), photometric changes (color jittering, random erasing *p* = 0.2), and Mixup regularization (α = 0.2, applied to 30% of batches). Three pretrained CNN backbone models process images in parallel: EfficientNet-B3 (1,536-dimensional features), DenseNet201 (1,920-dimensional features, growth rate *k* = 32), and ResNet101 (2,048-dimensional features). Each model share identical custom classification head with progressive dropout rates (0.65 → 0.55 → 0.45) and batch normalization.


FC(512)→FC(256)→FC(7)

Softmax normalized predictions are ensembled through weighted soft voting to obtain the final seven class predictions along with the confidence scores.


$$\left(w_{\text{Eff}},w_{\text{Dense}},w_{\text{Res}}\right)=\left(0.40,0.40,0.20\right)$$

Three XAI methods produce visual explanations after prediction. Grad-CAM with architecture-specific target layers (conv_head, denseblock4, layer4), LIME with SLIC segmentation (50 segments, κ = 10, σ = 1, 2,000 perturbed samples, top 20 features, *w*_min_ = 0.01), and OSA with systematic patch occlusion (*p* = 28, *s* = 14, gray value 128). TMAI validates the quality of explanations by using automated lesion segmentation in HSV and LAB color spaces, ensuring that the explanation concentrates on clinically relevant lesion areas. The full inference pipeline is described in Algorithm 5.

Algorithm 5 Proposed ensemble-based skin lesion classification with XAI and TMAI trustworthiness validation. 
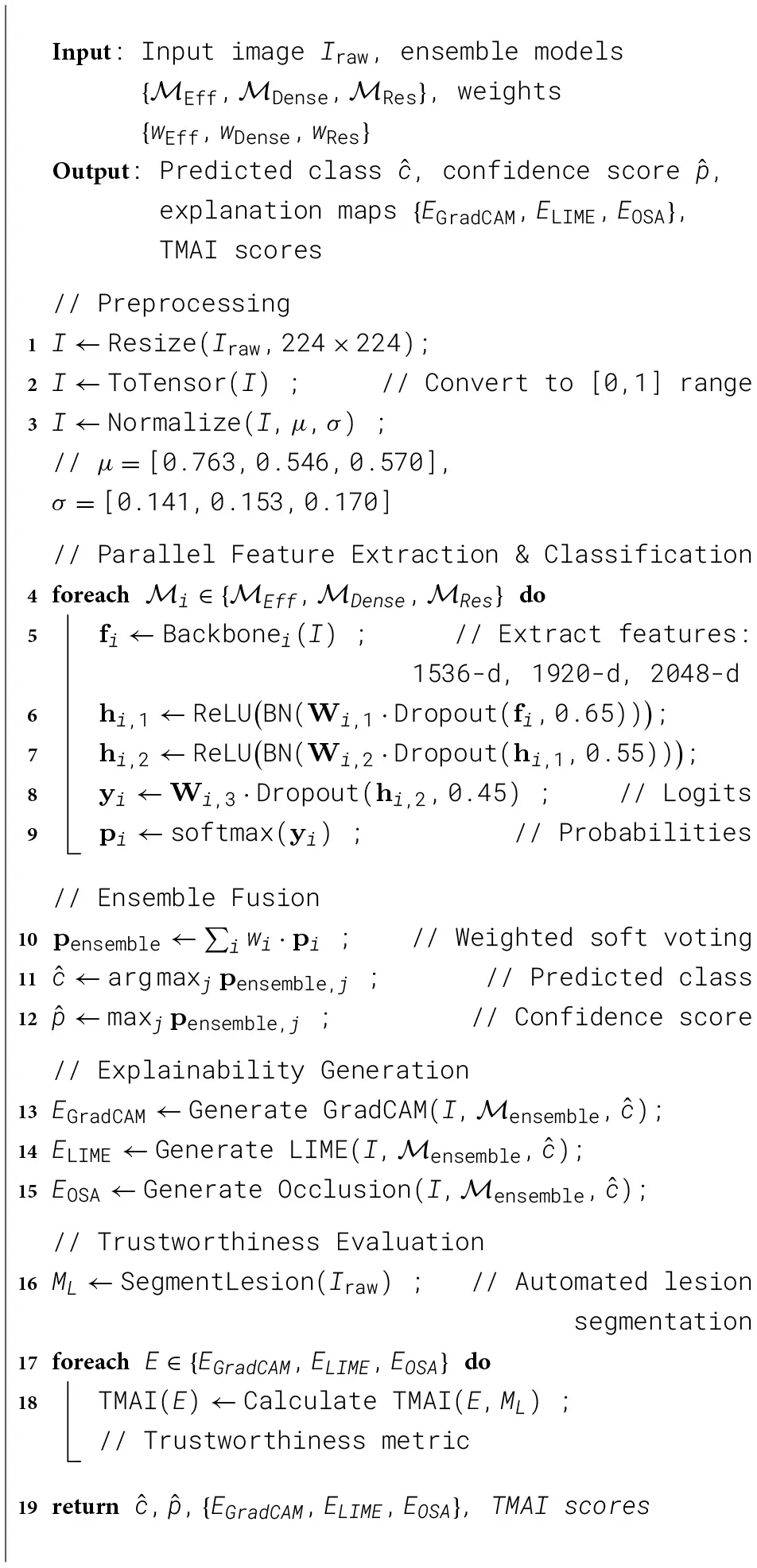

The algorithm follows four stages: preprocessing, parallel inference with three models, weighted ensemble fusion, and explainability generation with TMAI validation. The modular architecture enables selective execution of XAI components according to clinical needs. The classification inference time per image is typically less than 100 ms with GPU hardware. The XAI methods, including LIME with 2,000 perturbed samples per image and OSA with systematic sliding-window occlusion (patch size *p* = 28, stride *s* = 14), require additional processing time beyond the classification inference and are intended for explainability analysis rather than real-time classification.

## 4 Results and discussion

In this section we present the experimental results of the proposed ensemble model using three XAI techniques. The evaluation results covers training convergence, classification performance, per-class performance, XAI validation using TMAI, ablation study, statistical analysis, cross validation analysis, generalizability evaluation on ISIC 2019 dataset, and comparison with state-of-the-art methods. The experiments were carried on HAM10000 dataset which is mentioned in Section 3 showing the effectiveness of the ensemble architecture as well as the reliability of the XAI.

### 4.1 Experimental setup

The experiments were performed on NVIDIA Tesla T4 GPU using PyTorch 2.10.0 for the effective training of the model. For measuring the evaluation metrics scikit-learn is used, timm for EfficientNet-B3, torchvision for ResNet101, and DenseNet201. The HAM10000 dataset was used throughout the experiment to maintain consistent evaluation. Data augmentation included geometric transformation (rotation, flipping, affine), photometric adjustment (color jittering, random erasing), and Mixup regularization (α = 0.2) which is applied for 30% of training batches. The experiments were performed using three different pre-trained CNN architectures which were further fine-tuned with custom classification heads using progressive dropout (0.65 → 0.55 → 0.45) and batch normalization to maintain training stability. The models were trained with a batch size of 32 up to 50 epochs. A fixed random seed of 10 was set for all experiments to ensure full reproducibility of the reported results. The process involves early stopping to prevent overfitting, stop the training process if there is no further improvement for 12 successive epochs (with minimum change in improvement of threshold: 0.0003, applied after epoch no 25). Focal Loss (α = 1, γ = 2.2) had been used for managing class imbalance by focusing more training on harder samples which were present. AdamW optimizer used with different learning rates for each model (EfficientNet-B3: 1 × 10^−4^, DenseNet201: 7.5 × 10^−5^, ResNet101: 5 × 10^−5^), weight decay of 1.5 × 10^−3^ and StepLR learning rate scheduler reduces learning rate by 0.7 for every 10 epochs. Gradient clipping (max norm 0.5) applied for maintaining stable training. The final ensemble combined predictions of all 3 models based on weighted soft voting, with weights (*w*_Eff_, *w*_Dense_, *w*_Res_) = (0.40, 0.40, 0.20) were assigned based on individual validation performance. Finally the model performance evaluated using accuracy, precision, recall, F1-score, and per-class AUC. Confusion matrices were used to analyse all misclassification patterns with weighted averaging to account class imbalance. Three different XAI methods (Grad-CAM, LIME, and Occlusion Sensitivity) were measured using the TMAI.

### 4.2 Training convergence

**Figure 4:**
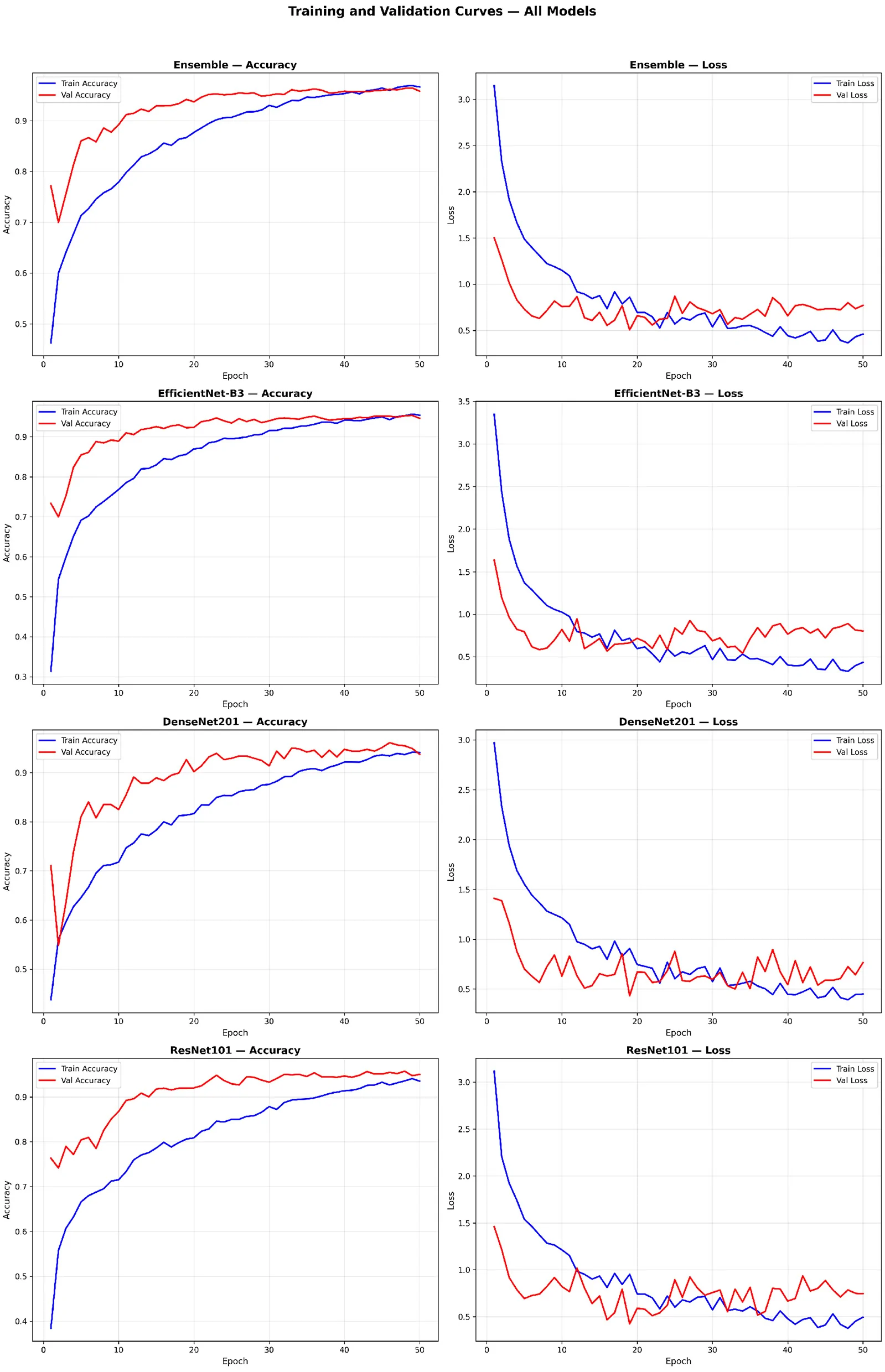
Training and validation performance comparison of individual models and the ensemble model.

Figure 4 shows the training behavior of the proposed ensemble model. The training accuracy rises from 40.81% in epoch 1 to 80% by epoch 15 and reaches 96.95% in the final epoch. Validation accuracy starts at 77.15% and crosses 90% by epoch 11, reaching a peak of 96.37% at epoch 49. Training loss decreases gradually from 3.84 to 0.408, and the validation loss drops from 1.50 to a best of 0.507 at epoch 19. The similar behavior of training and validation curves after epoch 20 indicates stable convergence without overfitting. EfficientNet-B3 converges quickly, reaching a final validation accuracy of 95.38% and validation loss of 0.807. DenseNet201 shows slightly unstable behavior during early training, improving to a final validation accuracy of 94.92% with a validation loss of 0.635. ResNet101 follows a slower but steady learning process, reaching a final validation accuracy of 94.74% with a validation loss of 0.744. The ensemble model achieves validation accuracy of 96.37%, precision of 96.25%, recall of 96.37%, and F1-score of 96.23%. The proposed ensemble model improves by 0.99 percentage points over the best individual model (EfficientNet-B3: 95.38%), validating the effectiveness of weighted soft voting with coefficients (0.40, 0.40, and 0.20).

### 4.3 Classification performance

Table 6 shows the overall classification performance metrics of all models. The ensemble model achieves the best performance, with 96.37% accuracy, 96.25% precision, 96.37% recall, 96.23% F1-score, and a macro-average AUC of 0.983, showing consistent and optimal performance across all seven diagnostic categories without biasing majority classes. Among all the individual models, EfficientNet-B3 achieves 95.38% accuracy, DenseNet201 achieves 94.92% accuracy, and ResNet101 achieves 94.74% accuracy. The ensemble model improves by 0.99 percentage points over the best individual model and maintains balanced, optimal precision-recall values, confirming that combining three diverse models through weighted soft voting provides reliable, consistent predictions. The ensemble model shows strong performance across all seven skin lesion categories. For clinically critical malignant lesions, the proposed model achieves 99.77% accuracy for MEL and perfect classification for BCC (100%, 35/35; 95% CI: 90.1%–100.0%), which is important for cancer screening, where missing a malignant case can have serious consequences. VASC (100%, 13/13; 95% CI: 77.2%–100.0%), while DF (87.50%, 7/8; 95% CI: 52.9%–97.8%), showing that Focal Loss (α = 1, γ = 2.2) successfully prevents the model from being biased toward majority classes. The balanced precision and recall values across all categories, including NV, BKL, and AKIEC, indicate that the model delivers strong performance, making it well suited for correctly detecting cancer cases and avoiding unnecessary biopsies. It is important to note that the perfect classification results (100%) observed for BCC and VASC are attributed to the effectiveness of class-specific augmentation and Focal Loss in handling minority classes. However, these results should be interpreted with caution given the limited number of validation samples for these classes.

**Table 6:** Overall classification performance metrics.

Model	Accuracy (%)	Precision (%)	Recall (%)	F1-score (%)	AUC
EfficientNet-B3	95.38	95.39	95.38	95.22	0.966
DenseNet201	94.92	94.73	94.92	94.75	0.974
ResNet101	94.74	94.52	94.74	94.51	0.971
**Ensemble**	**96.37**	**96.25**	**96.37**	**96.23**	**0.983**

Bold values indicate the best performance for each metric.

### 4.4 Confusion matrix analysis

**Figure 5:**
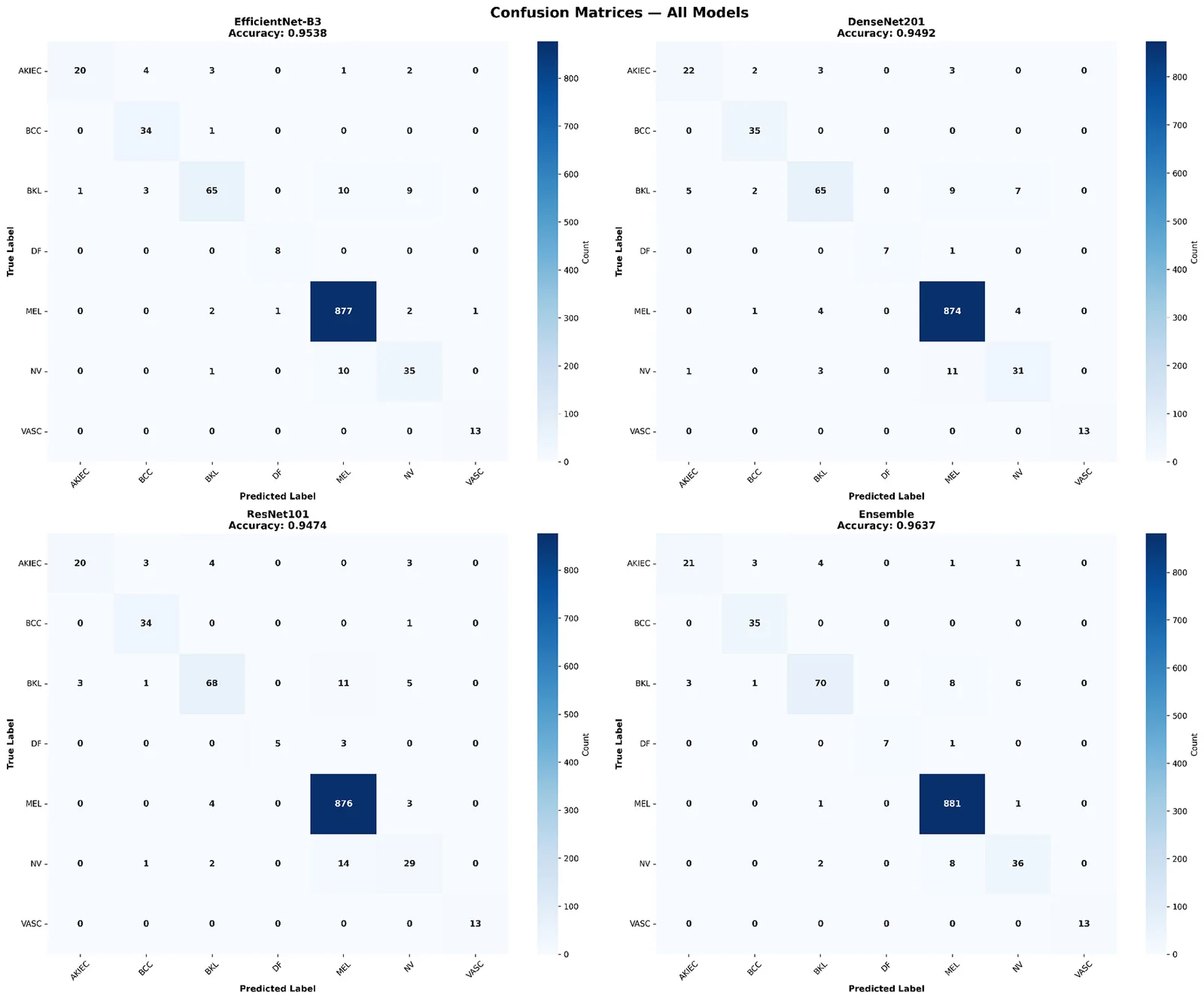
Confusion matrices comparing classification performance across seven skin lesion categories.

Figure 5 illustrates the confusion matrices for all models across the seven diagnostic categories, showing detailed classification patterns and the performance gains achieved by the ensemble model. All individual models show strong diagonal dominance with accuracies of 95.38% (EfficientNet-B3), 94.92% (DenseNet201), and 94.74% (ResNet101), while the ensemble model achieves a higher accuracy of 96.37% by correcting errors made by the individual models. The ensemble model achieves perfect classification (100%) for two categories, namely BCC (100%, 35/35; 95% CI: 90.1%–100.0%), VASC (100%, 13/13; 95% CI: 77.2%–100.0%). BCC and VASC sustain perfect scores across all individual models. DF achieves (87.50%, 7/8; 95% CI: 52.9%–97.8%) in the ensemble model, improving from 62.50% in ResNet101 to 87.50%, demonstrating the effectiveness of ensemble fusion for minority classes.MEL is the most clinically important category and achieves 99.77% accuracy in the ensemble model, with individual models attaining 99.32% (EfficientNet-B3), 98.98% (DenseNet201), and 99.21% (ResNet101). In the ensemble model, all the minority classes also show clear improvement. AKIEC class improves from 66.67% (EfficientNet-B3 and ResNet101) and 73.33% (DenseNet201), and the ensemble model achieves 70.00%. BKL shows good improvement with the ensemble model, which achieves 79.55%, better than the individual models: 73.86% (EfficientNet-B3), 73.86% (DenseNet201), and 77.27% (ResNet101). The ensemble model gives 78.26% accuracy for NV, which is better than all individual models (EfficientNet-B3: 76.09%, DenseNet201: 67.39%, ResNet101: 63.04%). The NV-MEL boundary indicates a challenge in dermoscopy, whereby benign moles and melanoma in early stages of growth present highly related visual features which are hard to differentiate even for dermatologists. This means the remaining errors are not due to limitations of the ensemble approach but to diagnostic difficulty that cannot be fully resolved by computational methods alone. BKL is most likely confused with NV and MEL because all three are pigmented skin lesions that have similar visual appearance on dermoscopy images. NV is confused with MEL mainly, which is the most clinically important error because it involves the boundary between a benign mole and a malignant tumor, and is genuinely difficult even for experienced dermatologists. AKIEC is confused with BCC and BKL, as expected, since all three can have similar rough, scaly surface textures and color patterns on dermoscopy. Importantly, the errors made by the model are not random; they consistently occur between categories that are known to be visually similar, which validates the model behavior because it makes mistakes in the same difficult areas where human experts also struggle.

### 4.5 Ensemble vs. individual models

The ensemble model achieves an accuracy of 96.37%, which is 0.99 percentage points higher than the best individual model performance (EfficientNet-B3: 95.38%), and the error is decreased from 4.62% to 3.63%. This improvement is achieved by combining the strengths of three models. EfficientNet-B3 applies compound scaling for efficient multi-scale feature extraction, DenseNet201 applies dense connectivity to preserve fine-grained spatial information across layers, and ResNet101 provides deep residual learning with skip connections to enable gradient flow. The weighted soft voting approach allocates the coefficients of (*w*_Eff_, *w*_Dense_, *w*_Res_) = (0.40, 0.40, 0.20) based on individual validation performance, where higher weights are assigned to EfficientNet-B3 and DenseNet201 while ResNet101 provides ensemble overall stability.

**Figure 6:**
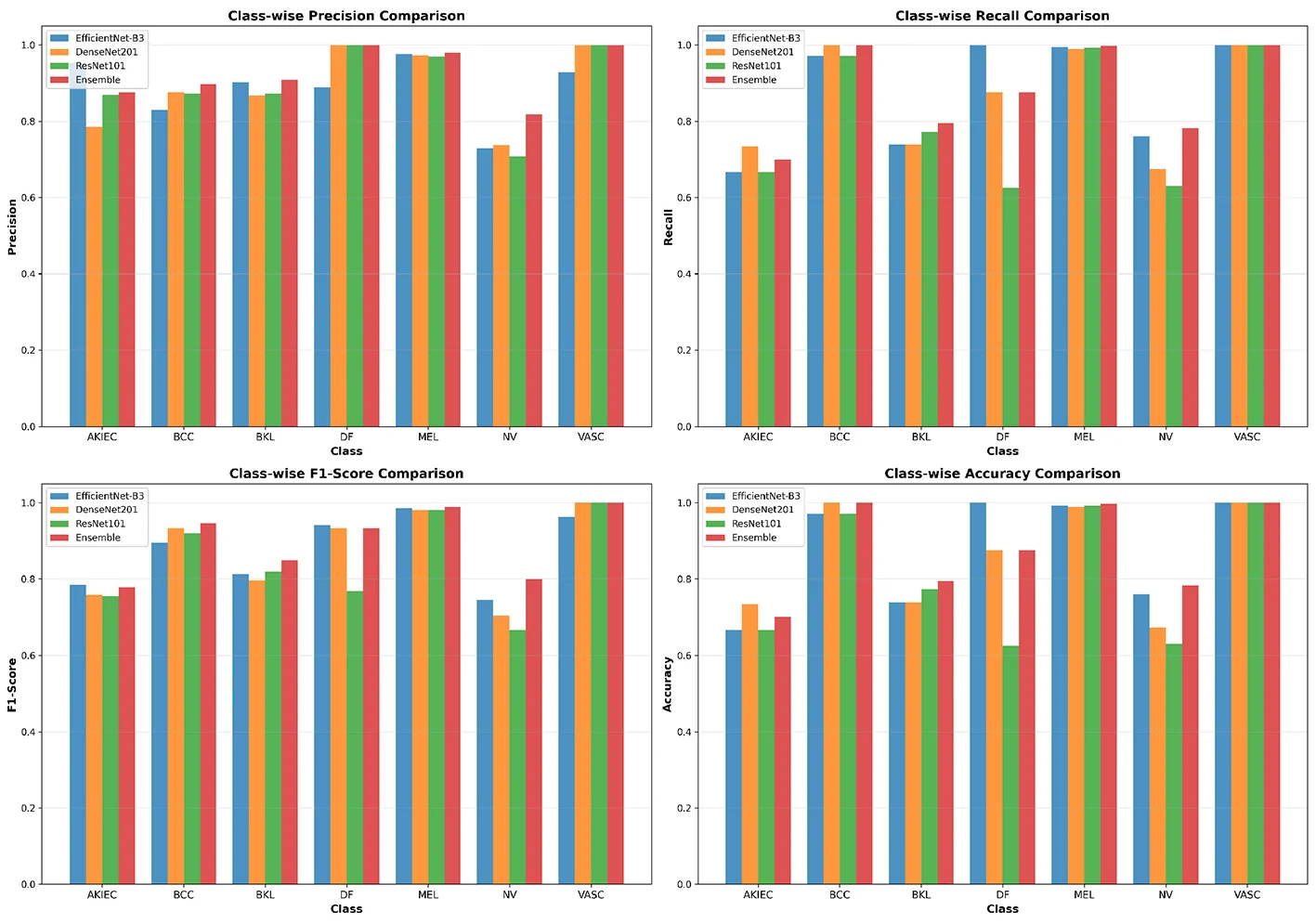
Class-wise performance comparison across individual models.

Figure 6 presents the class-wise performance for all models, highlighting which diagnostic classes benefit most from ensemble integration. AKIEC shows F1-score values of approximately 0.75–0.78 across individual models, with the ensemble achieving approximately 0.78. BKL shows improvement with F1-score ranging from approximately 0.80–0.82 in individual models to approximately 0.85 in the ensemble. NV shows improved precision from approximately 0.70–0.74 in individual models to approximately 0.82 in the ensemble. VASC achieves perfect scores (1.0) across all evaluation metrics, while DF achieves (87.50%, 7/8; 95% CI: 52.9%–97.8%) in the ensemble model, showing improvement over ResNet101 (62.50%). BCC achieves consistently high F1-scores of approximately 0.93–0.94 across all models. MEL maintains very high, consistent performance (F1: ≈0.99) across all models, with improved performance in the ensemble. Overall, the ensemble method provides the strongest and most balanced performance across classes, with notable gains observed for BKL and NV while maintaining excellent performance for the remaining categories.

### 4.6 ROC curve analysis

**Figure 7:**
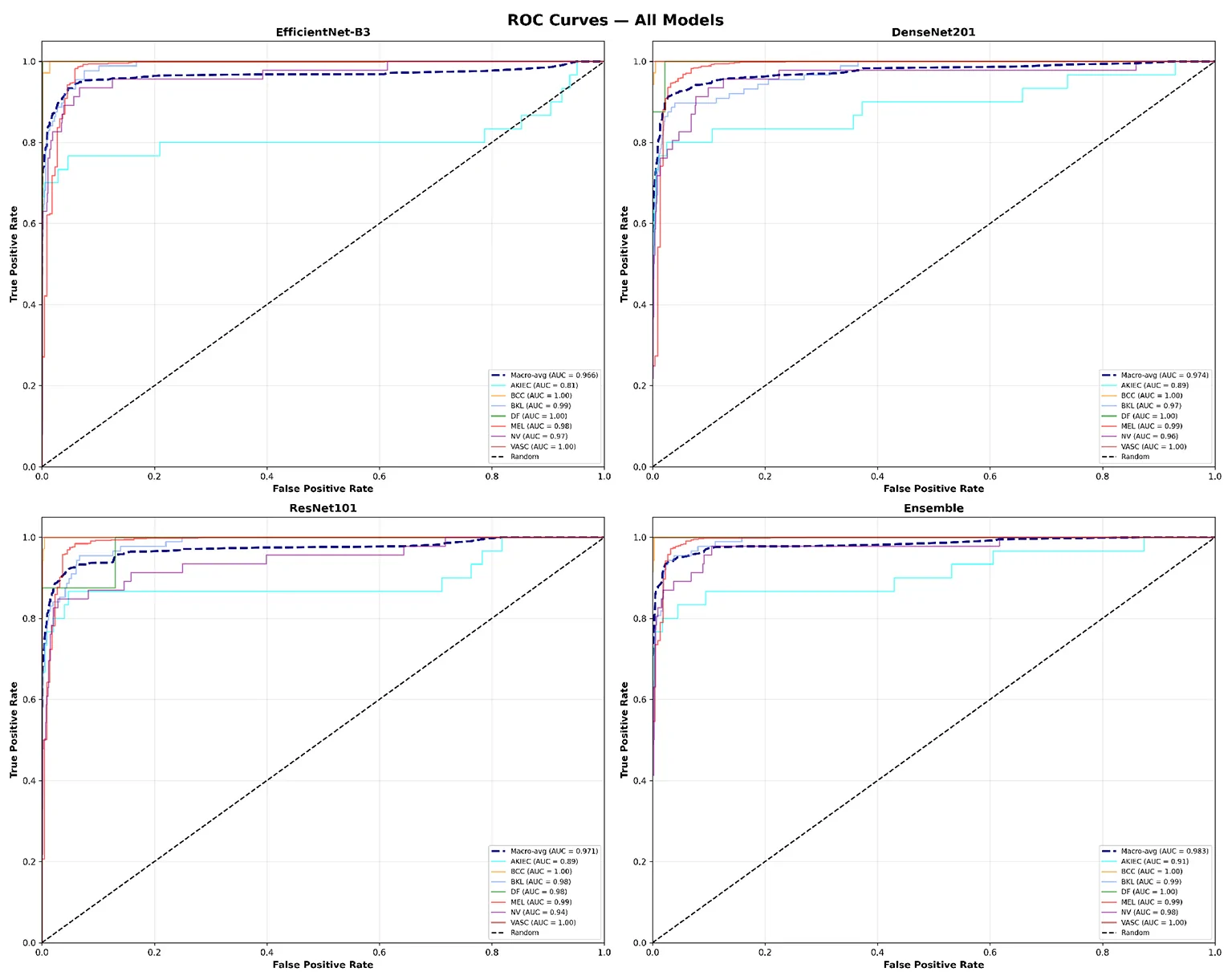
ROC curves for all seven diagnostic categories across individual models and ensemble.

Figure 7 shows ROC curves for all models across seven diagnostic categories. The ensemble model achieves a macro-average AUC of 0.983, outperforming all individual models (EfficientNet-B3: 0.966, DenseNet201: 0.974, ResNet101: 0.971). This improvement shows that the ensemble model has stronger discriminative capability across all diagnostic categories than any single backbone model. The ensemble model achieves AUC = 1.00 for BCC, DF, and VASC, indicating perfect discrimination for these categories. BKL and MEL achieve AUC = 0.99, while NV achieves AUC = 0.98. The perfect discrimination for BCC is particularly significant for clinical screening, as it indicates the model can completely separate positive and negative cases for this malignant category. AKIEC, the most challenging minority class, improves from 0.81 (EfficientNet-B3) and 0.89 (ResNet101) to 0.91 with the ensemble model, showing that the ensemble effectively addresses class imbalance by leveraging the complementary strengths of the three backbone models. The consistently high AUC values across all categories, including minority classes such as DF, VASC, and AKIEC, confirm that the combination of Focal Loss (α = 1, γ = 2.2), class-specific augmentation, and ensemble fusion effectively handles severe class imbalance. The ensemble model achieves a macro-average AUC of 0.983, exceeding the clinically recommended threshold of 0.95 for computer-aided diagnosis systems, thereby confirming its strong diagnostic reliability.

### 4.7 Feature space visualization

**Figure 8:**
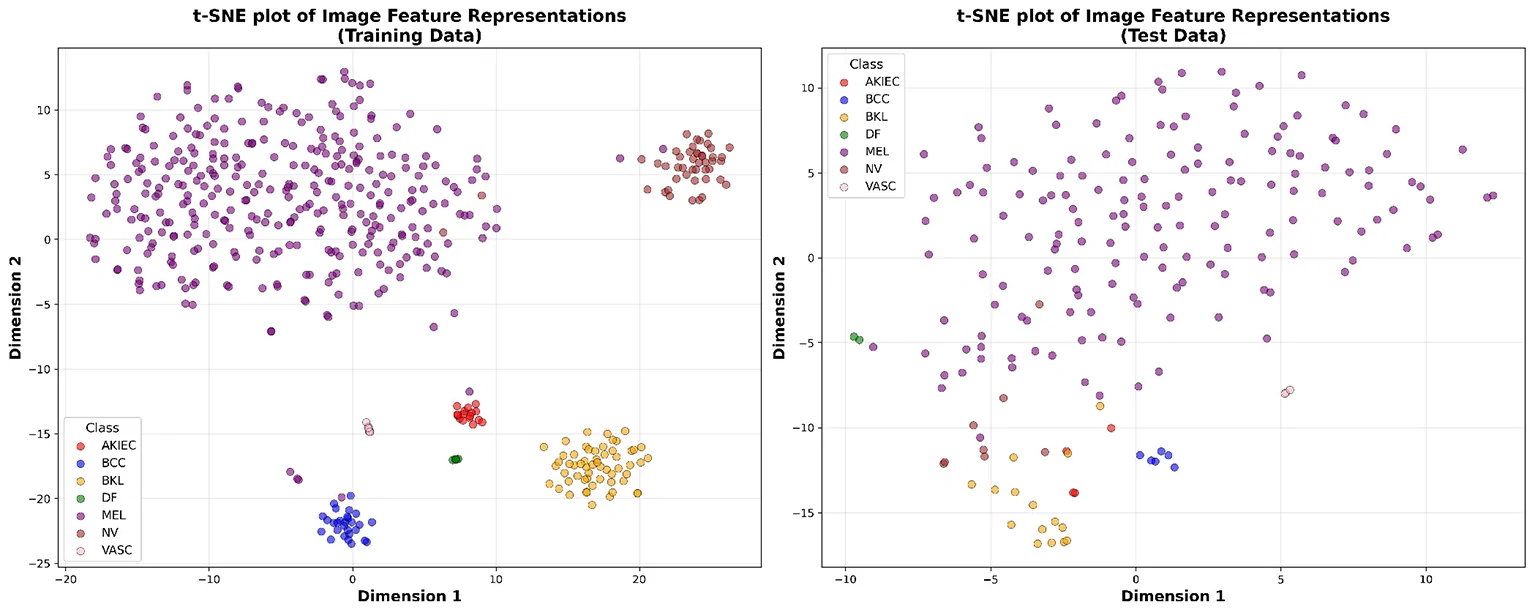
t-SNE visualization of ensemble model feature representations for training data **(left)** and validation data **(right)**.

Figure 8 presents t-SNE visualizations of the features extracted from the ensemble model from the training and validation sets. The 2D plots illustrate how well the features are separated, the relationships within the different classes, and how well the model generalizes across the seven diagnostic classes. The visualization of the training data reveals well-defined clusters for various lesion types, confirming that the model has learned effective distinguishing features. DF forms a compact, well-separated cluster, reflecting a unique structure that clearly distinguishes it from pigmented lesions. BCC occupies a different region, indicating that the model successfully represents its characteristic pearly, translucent appearance unique to this malignant non-melanoma category. BKL clusters in the lower-right region with moderate spacing between points indicating that the model learns useful features but also shows some variation within the class. VASC forms a few but distinct clear points, showing it is well separated from pigmented categories based on its color-specific features, even with fewer training samples. AKIEC forms a relatively compact cluster, indicating that the model has learned distinguishable features for this pre-cancerous class despite its varied appearance. Partial overlap is observed among MEL, NV, and BKL, reflecting similarities among pigmented lesions. The MEL cluster is more spread out, showing high variation, as melanoma can appear in different forms, and the NV cluster is more compact, showing a more consistent benign appearance. The validation visualization shows more overlap between classes than in training, especially among pigmented lesions (MEL, NV, and BKL), which is a challenge when dealing with unseen samples. Nevertheless, the validation accuracy of 96.37% confirms that the model maintains strong classification performance despite reduced feature-space separation. Minority classes (DF, VASC, and AKIEC), even with fewer samples, show identifiable clusters, indicating that class-weighted Focal Loss and data augmentation helped prevent the model from focusing only on majority classes. The continued overlap between MEL and NV in validation indicates a real classification challenge, similar to clinical practice, where even expert dermatologists have difficulty distinguishing between atypical nevi and early melanoma. The feature space supports the ensemble approach: well-separated classes (BCC, DF, and VASC) achieve high accuracy, while overlapping classes benefit from ensemble voting to handle difficult cases. The similar cluster patterns in both training and validation indicate that the model has learned general features rather than memorizing the training data, supporting the robustness and generalization capability of the proposed framework.

### 4.8 Explainability and trustworthiness

While the ensemble model achieves 96.37% classification accuracy, clinical deployment of deep learning systems requires transparent decision-making methods so that the physicians can verify and trust the results. Black-box predictions, regardless of accuracy, are not sufficient in medicine, as diagnostic decisions must be interpretable, auditable, and aligned with clinical standards. To address this, we combine three complementary XAI techniques that visualize the model's decision-making from different perspectives: Grad-CAM, LIME, and OSA.

#### 4.8.1 Gradient-weighted class activation mapping analysis

**Figure 9:**
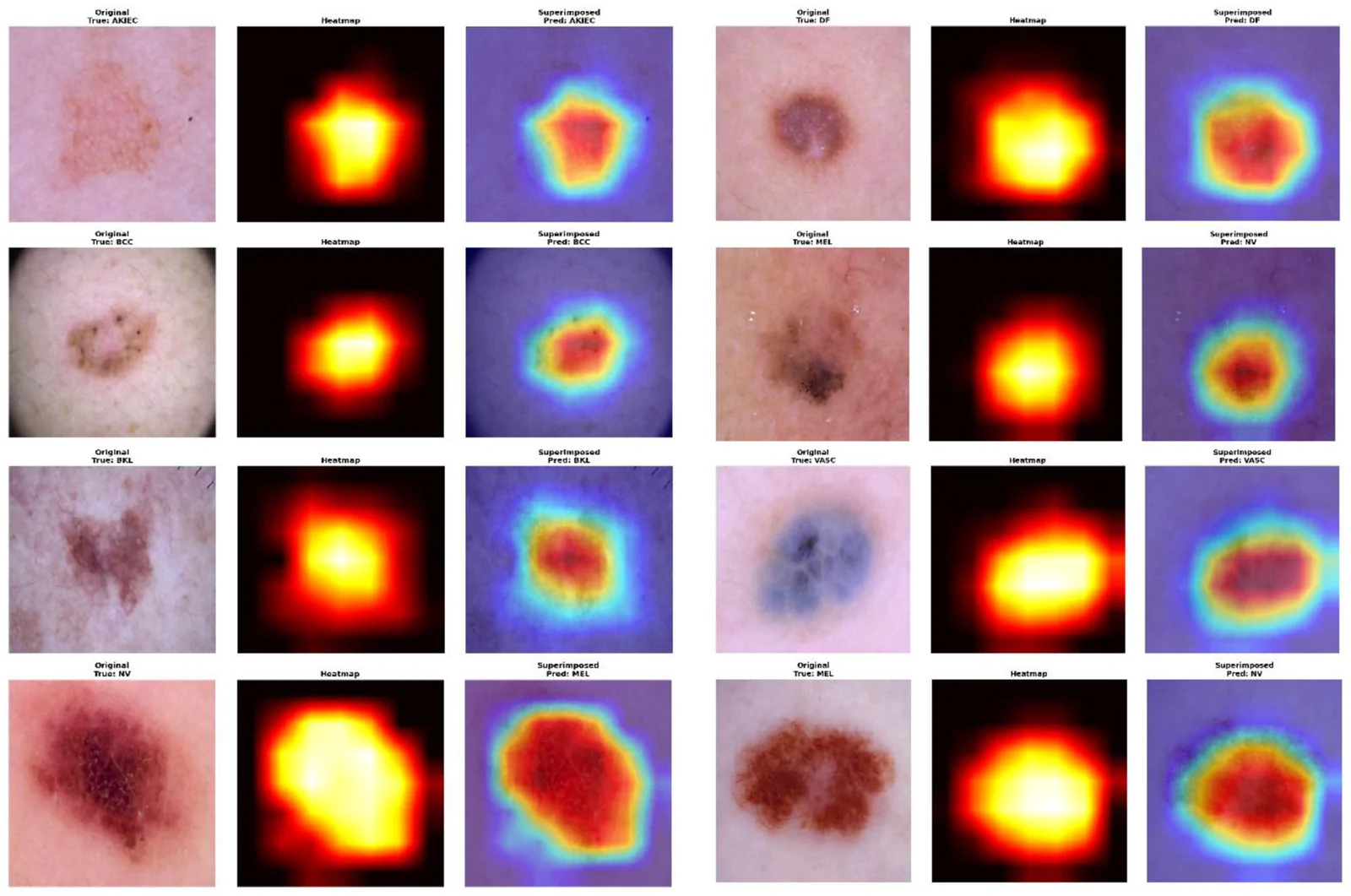
Ensemble Grad-CAM visualization showing weighted combination of attention maps.

Figure 9 presents Grad-CAM visualizations for representative samples from the HAM10000 dataset, highlighting the regions that contribute most to the weighted ensemble model's classification decisions. Grad-CAM generates class-discriminative heatmaps using gradients from the final convolutional layers, where warmer colors (yellow, orange, and red) indicate regions of higher importance, while cooler colors (blue and purple) represent regions of lower importance. For each lesion sample, the figure shows the original dermoscopic image, the corresponding Grad-CAM heatmap, and a superimposed visualization with the attention map overlaid on the original image. The visualizations demonstrate that the ensemble consistently focuses on clinically relevant lesion regions across different skin lesion categories. High-activation areas are predominantly concentrated within the lesion boundaries, while surrounding healthy skin receives minimal attention. For pigmented lesions such as MEL and NV, the model emphasizes regions exhibiting color variation, irregular pigmentation, and structural asymmetry. Similarly, for BKL, BCC, AKIEC, DF, and VASC, the attention maps are localized around diagnostically significant lesion structures rather than background artifacts. The superimposed heatmaps further confirm accurate lesion localization, indicating that the model relies on meaningful dermatological characteristics for classification. These findings demonstrate the interpretability of the proposed framework and provide evidence that the ensemble's predictions are driven by clinically relevant features, thereby enhancing the transparency, reliability, and potential clinical applicability of the system.

#### 4.8.2 Occlusion sensitivity analysis

**Figure 10:**
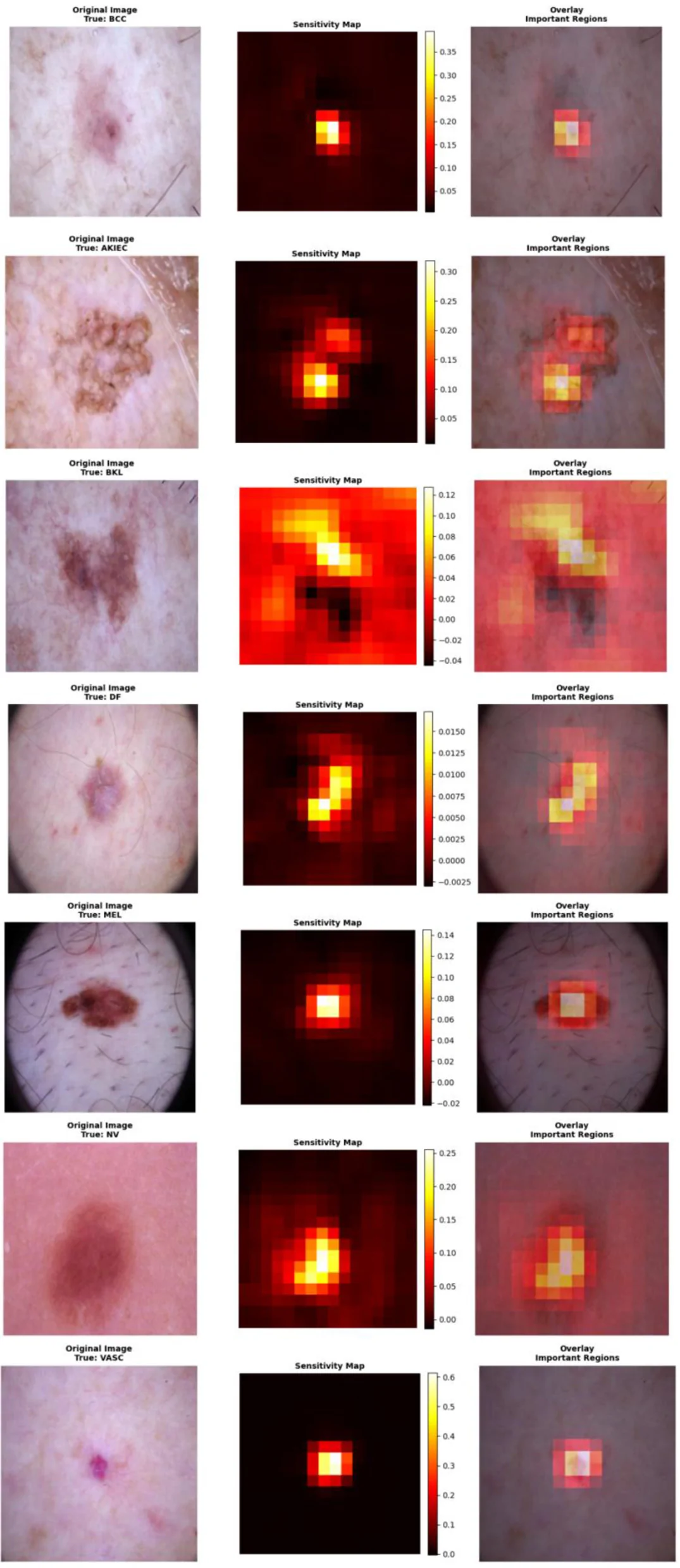
Occlusion sensitivity analysis for seven diagnostic categories showing original images **(left)**, sensitivity heatmaps **(center)**, and overlays **(right)**.

Figure 10 depicts OSA results across all diagnostic categories of HAM10000, which provides perturbation-based explanations that are independent of the model architecture. OSA systematically covers the image regions with gray patches (size *p* = 28 pixels, stride *s* = 14 pixels, intensity 128) and measures the model's confidence drop, directly capturing each region's importance in making the decision. Each row shows the original image (left), the sensitivity heatmap (center), and an overlay highlighting important regions (right). The sensitivity maps show clear lesion localization in all the classes, with highly important regions (bright yellow-white areas) focused within the lesion boundaries. VASC shows a high peak sensitivity (~0.6), indicating that the model clearly identifies the unique red vascular structure. BCC (~0.35) and AKIEC (~0.30) show strong activation in the central lesion area. MEL (~0.14) and NV (~0.20) show focused activation on the pigmented lesion regions. BKL (~0.12) and DF (~0.0150) show low sensitivity values because their visual features are subtle, not because of poor localization. The sensitivity scores range from DF (~0.0150) to VASC (~0.6), confirming that the model focuses on clinically relevant lesion features rather than background artifacts; combining GRAD-CAM and LIME further validates the model's explainability from multiple perspectives.

#### 4.8.3 Local interpretable model-agnostic explanations analysis

**Figure 11:**
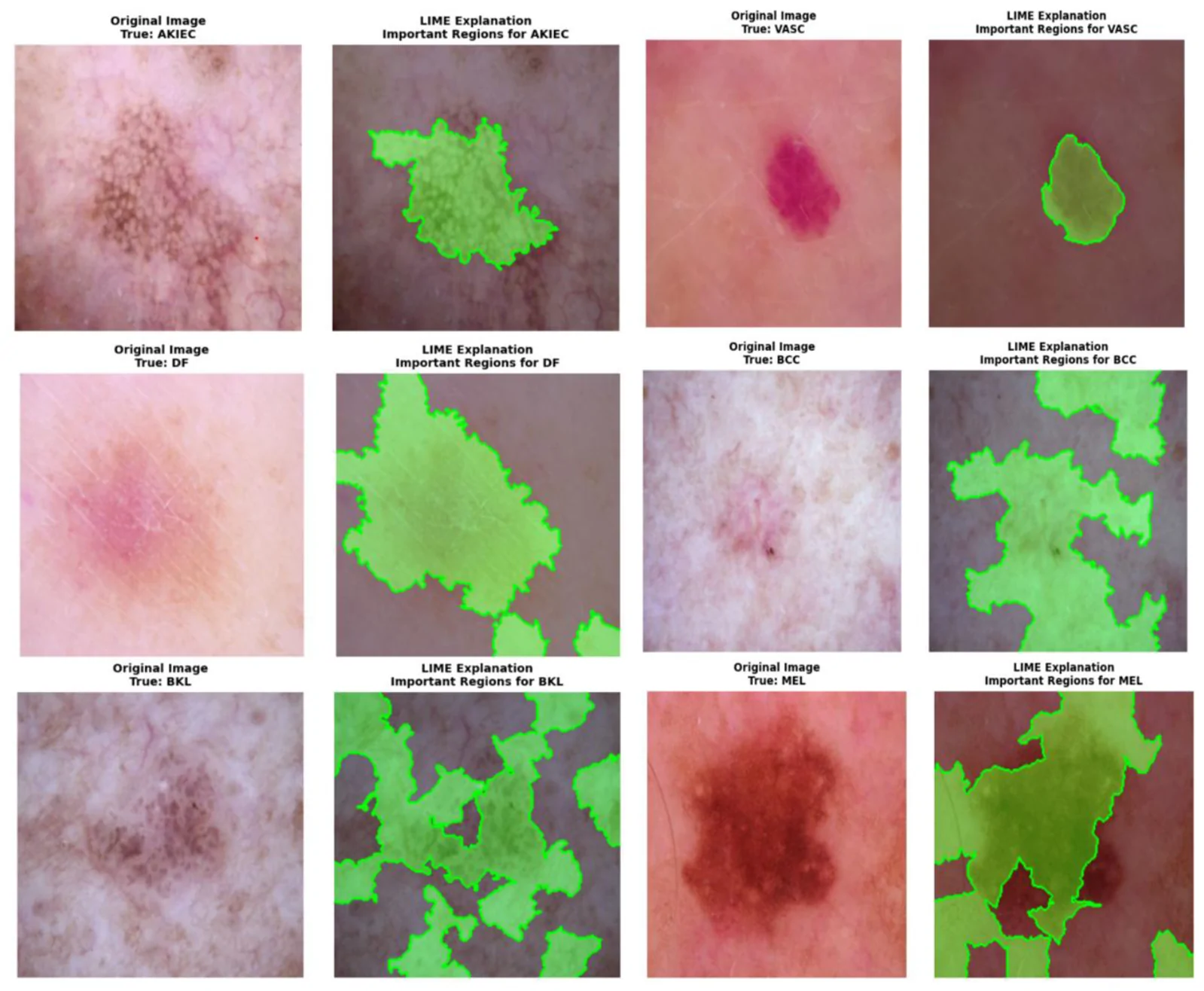
LIME explanations showing important regions for different diagnostic categories.

Figure 11 shows LIME visualizations for HAM10000 skin images across several classes, providing superpixel-level explanations using model-agnostic perturbation analysis. Here, the LIME partitions each input image into 50 superpixel regions by using SLIC segmentation (compactness κ = 10, smoothing σ = 1), generates 2,000 perturbed versions by randomly hiding the regions, and afterward extracts the most influential superpixels by considering a minimum weight *w*_min_ = 0.01), highlighted in green to indicate the regions that are important for disease classification. The LIME explanations show clear lesion localization across several diagnostic classes. VASC shows the greatest localization, with a narrow green region around red vascular structures. BKL exhibits the most scattered pattern because of the varied appearance of the lesion, where green regions appear in different parts of the lesion. AKIEC and DF show large green regions in the center of the lesion area. BCC displays several smaller green regions corresponding to its complex surface features, and also, MEL displays a large central green region with some extensions at the border of the lesion. In all the cases discussed, the green regions appropriately identify the important region of the lesion and ignore the background, supporting the findings from Grad-CAM and Occlusion Sensitivity and confirming that the model focuses on the right features for diagnosis.

#### 4.8.4 Trustworthiness evaluation

**Figure 12:**
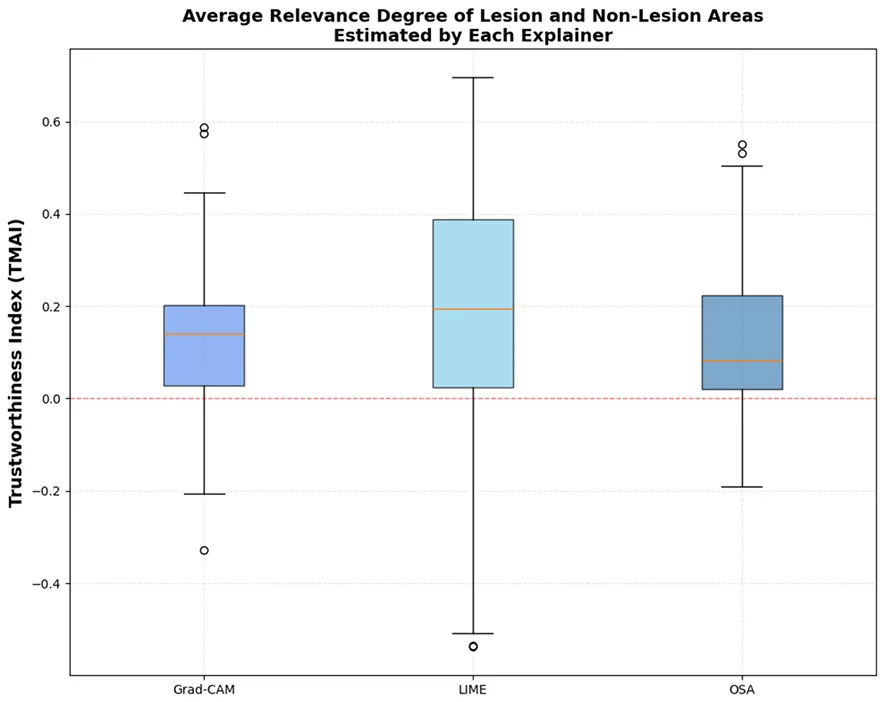
TMAI comparison across three explainability methods.

Figure 12 shows the results of the TMAI validation indicating the comparison of the explanation quality of Grad-CAM, LIME, and OSA. TMAI quantifies whether explanations are concentrated in lesion areas rather than background, using automatic lesion segmentation in HSV and LAB color spaces. Positive TMAI values indicate lesion-focused (trustworthy) explanations, whereas negative values indicate background-focused (untrustworthy) explanations. All three XAI methods have positive mean and median TMAI scores, indicating that the explanations are always focused on the lesion areas. LIME, on the other hand, achieves the highest trustworthiness (mean: 0.1457, median: 0.1952) and precision due to superpixel-based region segmentation, but also has the highest variability (std: 0.2689) because it is sensitive to irregular lesion boundaries. Grad-CAM has the most stable performance (mean: 0.1245, median: 0.1404, std: 0.1631) and a compact distribution and few outliers. OSA achieves a mean TMAI of 0.1324 but a lower median TMAI (0.0824), driven by its coarser patch-based localization (*p* = 28, *s* = 14), which distributes sensitivity over a wider area. The moderate TMAI values indicate the complexity of the dermoscopic images, where important diagnostic features can extend beyond the actual lesion border to nearby areas such as erythema, regression patterns, and border characteristics. Occasional negative TMAI values occur as outliers, reflecting complex dermoscopic cases with unclear lesion margins or poor contrast between the lesion and surrounding skin, which are difficult to assess even for expert dermatologists. Positive TMAI scores for all methods and minority classes indicate that the classification decisions rely on clinically relevant features, validating the trustworthiness of the ensemble model.

### 4.9 Comparison with state-of-the-art methods

Table 7 presents the comparison of the proposed ensemble framework with other existing methods. For a fair evaluation, we focused on methods tested on the HAM10000 dataset; we also included other methods to provide general context. The symbol “–” indicates values were not provided in the study.

**Table 7:** Comparison with state-of-the-art methods.

References	Method	Dataset	Acc	Pre	Rec	F1	AUC	XAI	TMAI
			(%)	(%)	(%)	(%)			
Groh et al. (9)	Physician-AI	Custom	33/69	–	–	–	–	No	No
Abohashish et al. (10)	LSTM+CNN	HAM10000	96.21	94.75	94.93	95.55	0.99	No	No
Cheng et al. (11)	MobileNet-MFS	ISIC-2019	87.30	87.32	87.51	87.30	0.9631	No	No
Brinker et al. (12)	Benchmark	MClass-D/ND	–	89.4	64.4	–	0.769	No	No
Haenssle et al. (13)	Inception V4	Dermoscopic	–	–	88.9	–	0.82	No	No
Moura et al. (14)	ABCD+CNN+MLP	Custom	94.90	94.8	94.8	–	–	No	No
Jaisakthi et al. (15)	EfficientNet-B6	ISIC-2019/2020	–	–	–	–	0.9681	No	No
Kawahara et al. (16)	Multi-task InceptionV3	Custom	74.2	65.3	61.4	–	0.86	No	No
Halder et al. (18)	Fuzzy Ensemble	HAM10000	95.14	–	–	–	–	Grad-CAM	No
Hammouda et al. (32)	U-Net + Pretrained Classifier	HAM10000	89.90	89.70	90.10	90.20	0.893	No	No
Murali and Mazumder (33)	DermaScanAI / SkinNet-HDA	HAM10000	93.21	92.19	91.12	91.65	0.962	Grad-CAM++, SHAP	No
Alhaj and Ozturk (34)	GC-ViT-E	HAM10000	92.81	90.61	90.03	90.29	0.9908	Grad-CAM	No
Xin et al. (35)	SkinTrans-ViT	HAM10000	94.30	94.10	–	–	0.987	Grad-CAM	No
**Proposed**	**Weighted ensemble**	**HAM10000**	**96.37**	**96.25**	**96.37**	**96.23**	**0.983**	**Grad-CAM** **+** **LIME** **+** **OSA**	**Yes**

Bold values indicate the best performance for each metric.

The proposed ensemble model achieves 96.37% accuracy and a macro-average AUC of 0.983. Importantly, this is the only framework that uses multi-method XAI validation with Grad-CAM, LIME, and OSA, with quantitative TMAI trustworthiness scores, addressing the critical gap in explainability validation in the literature. Furthermore, most studies focus on binary classification of melanoma vs. non-melanoma, but in real time doctors need to identify all seven skin disease types. The proposed framework demonstrates strong clinical relevance by achieving high sensitivity for malignant lesions such as MEL and BCC, which are critical for early cancer detection. However, clinical deployment requires further validation through dermatologist evaluation and testing on multi-center datasets to ensure reliability across diverse patient populations and imaging equipment.

### 4.10 Ablation study

To evaluate the contribution of each proposed component, we conducted a systematic ablation study on the HAM10000 dataset. We designed four progressive experimental configurations by incrementally adding key components: Focal Loss, class-specific augmentation, and Mixup augmentation. Each configuration was trained independently using EfficientNet-B3 as the sole backbone model, while the full proposed model employs a weighted soft-voting ensemble of EfficientNet-B3, DenseNet201, and ResNet101 with all components active. The results are summarized in Table 8.

**Table 8:** Ablation study results showing the contribution of each component.

Experiment	Accuracy (%)	F1 score (%)	AUC
Baseline	92.11	91.66	0.9740
+Focal Loss	92.48	92.12	0.9687
+Class Aug	91.48	91.04	0.9453
+Mixup	91.75	91.40	0.9543
**Full model (proposed)**	**96.37**	**96.23**	**0.983**

Bold values indicate the best performance for each metric.

As shown in Table 8, the baseline configuration achieved a validation accuracy of 92.11% with a weighted F1-score of 91.66% and a macro AUC of 0.9740. Adding Focal Loss improved accuracy to 92.48% and F1-score to 92.12%, demonstrating its effectiveness in handling class imbalance by assigning higher penalties to misclassified minority samples. While class-specific and Mixup augmentation show only marginal gains when applied to a single backbone model, their true effectiveness emerges within the full ensemble framework. This is consistent with findings in the literature, where augmentation strategies tend to yield greater benefits when combined with heterogeneous ensemble architectures rather than standalone models. The full proposed model, incorporating all components along with weighted soft voting ensemble fusion, achieved the highest accuracy of 96.37%, weighted F1-score of 96.23%, and macro AUC of 0.983, representing a substantial improvement of 4.26% over the single-model baseline. The baseline configuration struggled with minority classes, achieving only 60.00% for AKIEC, 50.00% for DF, and 41.30% for NV. Conversely, the full proposed model significantly improved performance across all classes, achieving 70.00% for AKIEC, 87.00% for DF, and 78.00% for NV, along with perfect classification (100%) for BCC and VASC and 99.77% for MEL. These results confirm that the combination of Focal Loss, class-specific augmentation, Mixup, and ensemble fusion works synergistically to address class imbalance and improve overall classification robustness. The ablation study therefore validates each design choice in the proposed framework and justifies selecting the full ensemble configuration as the final model.

### 4.11 Statistical analysis

To rigorously validate the reported performance improvements, a comprehensive statistical analysis was conducted on the proposed ensemble framework. Bootstrap confidence intervals were computed over 1,000 resampling iterations to assess the stability of the reported metrics. As presented in Table 9, the proposed ensemble achieved an accuracy of 96.37% with a 95% confidence interval of [95.38%, 97.55%] and a weighted F1-score of 96.23% with a 95% confidence interval of [95.14%, 97.47%]. The narrow confidence interval ranges confirm the stability and consistency of the proposed framework. The Wilcoxon signed-rank test was used to statistically validate whether the performance improvements achieved by the proposed ensemble over individual models are statistically significant. As presented in Table 10, the ensemble significantly outperforms EfficientNet-B3 (*p* = 0.001341), DenseNet201 (*p* = 0.001595), and ResNet101 (*p* = 0.000145), with all p-values well below the significance threshold of 0.05. These results confirm that the performance improvements are statistically meaningful and not due to random chance. The ensemble weight configuration was further validated through an ablation study presented in Table 11 demonstrating that the proposed weight configuration (EfficientNet-B3: 0.40, DenseNet201: 0.40, ResNet101: 0.20) achieves the highest accuracy of 96.37% compared to all alternative weighting strategies.

**Table 9:** Bootstrap confidence intervals for the proposed ensemble framework on the HAM10000 dataset.

Metric	Value	CI lower	CI upper
Accuracy	96.37%	95.38%	97.55%
Weighted F1	96.23%	95.14%	97.47%

**Table 10:** Wilcoxon signed-rank test results comparing the proposed ensemble against individual models on the HAM10000 dataset.

Comparison	*p*-value	Significant
Ensemble vs. EfficientNet-B3	0.001341	Yes
Ensemble vs. DenseNet201	0.001595	Yes
Ensemble vs. ResNet101	0.000145	Yes

**Table 11:** Ensemble weight ablation study comparing different weight configurations on the HAM10000 dataset.

Configuration	Accuracy (%)
Equal (0.33/0.33/0.33)	96.01
Alt-1 (0.50/0.30/0.20)	96.19
Alt-2 (0.60/0.20/0.20)	95.56
**Proposed (0.40/0.40/0.20)**	**96.37**

Bold values indicate the best performance for each metric.

### 4.12 Cross-validation analysis

To further validate the consistency and reliability of the reported results, we conducted a comprehensive 3-fold cross-validation on the HAM10000 dataset, maintaining strict lesion-level partitioning across all folds to prevent lesion-level overlap between training and validation sets. Table 12 presents the cross-validation results across all three folds. The narrow standard deviation across all metrics confirms the stability and consistency of the proposed ensemble framework across different data partitions. The mean accuracy of 95.56% ± 0.32% is consistent with the reported validation accuracy of 96.37%, confirming that the reported results are reliable. The hyperparameters used in 3-fold cross-validation were identical to those established during the original model development. No hyperparameter tuning was performed during cross-validation, ensuring an independent assessment of the fixed model configuration.

**Table 12:** 3-fold cross-validation results on the HAM10000 dataset.

Metric	Fold 1	Fold 2	Fold 3
Accuracy (%)	95.76	95.81	95.10
Precision (%)	95.73	95.87	94.96
Recall (%)	95.76	95.81	95.10
F1-score (%)	95.69	95.78	95.00
AUC	0.9771	0.9819	0.9756
Mean			
Accuracy (%)	95.56 ± 0.32		
Precision (%)	95.52 ± 0.40		
Recall (%)	95.56 ± 0.32		
F1-score (%)	95.49 ± 0.35		
AUC	0.9782 ± 0.0027		

### 4.13 Generalizability evaluation on ISIC 2019 dataset

**Figure 13:**
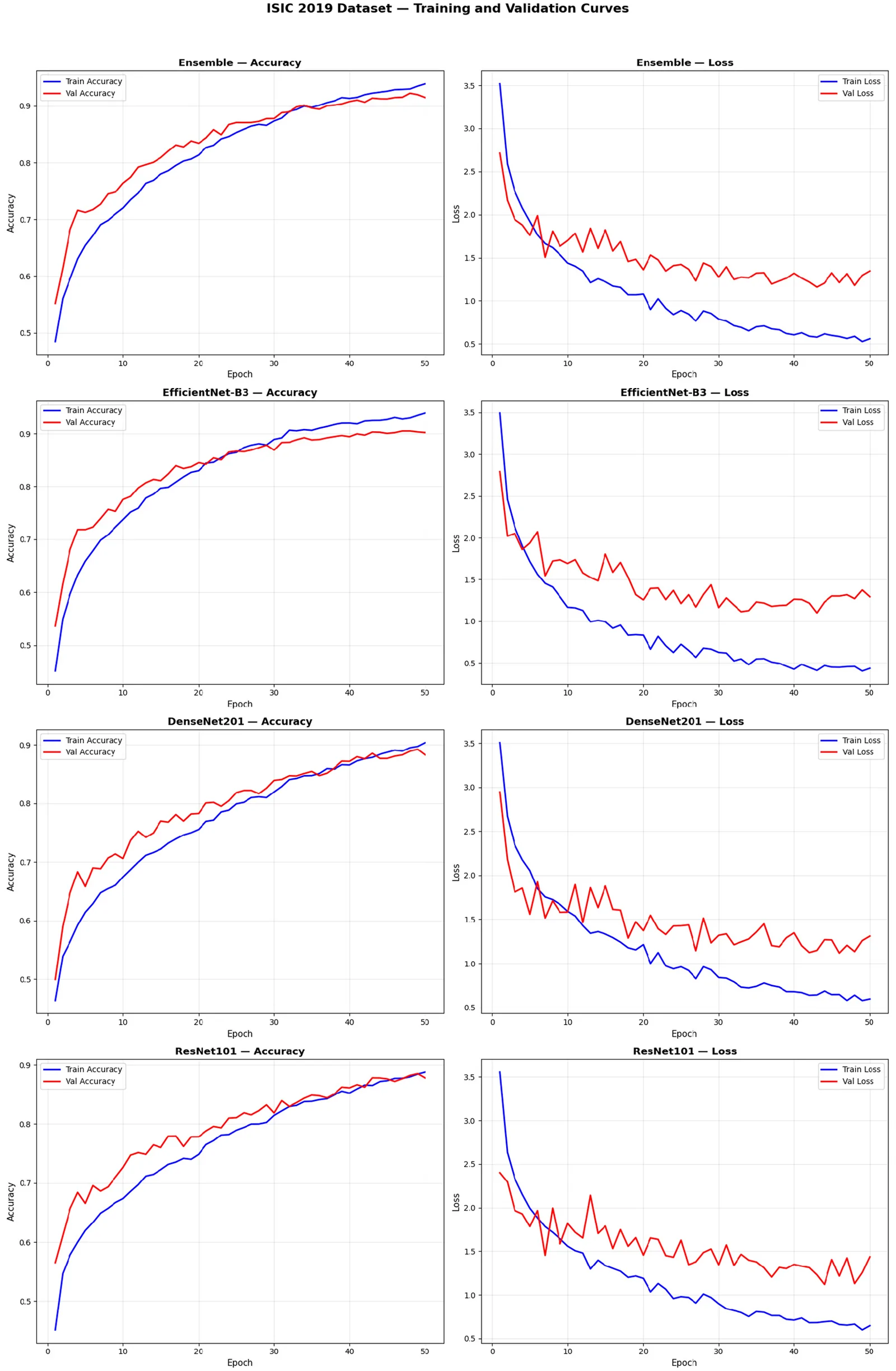
Training and validation curves for all models on the ISIC 2019 dataset.

**Figure 14:**
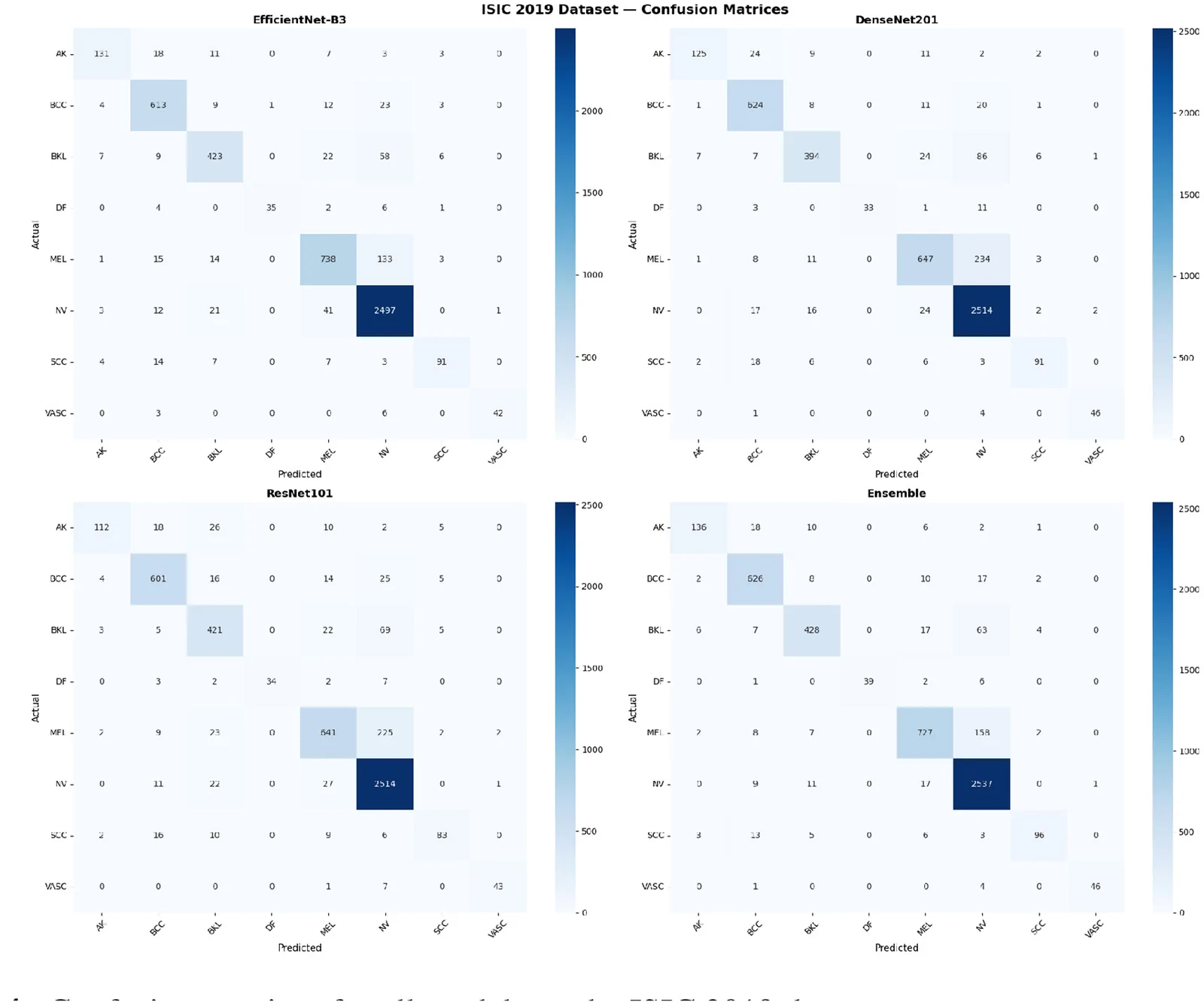
Confusion matrices for all models on the ISIC 2019 dataset.

**Figure 15:**
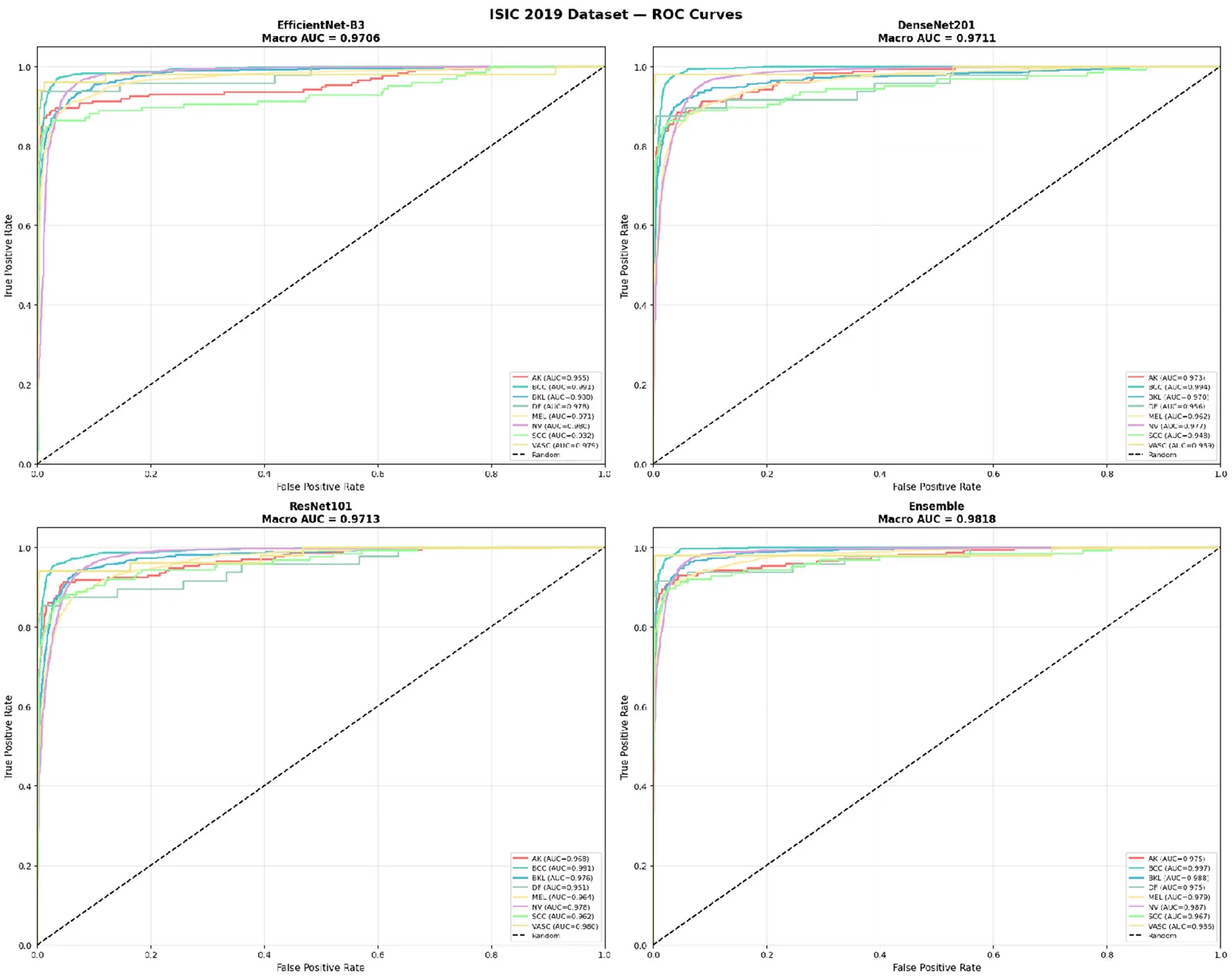
ROC curves for all models on the ISIC 2019 dataset.

To assess the proposed model's generalizability, we independently retrained the framework on the ISIC 2019 dataset, comprising 25,331 dermoscopic images across eight diagnostic categories. We applied the same ensemble architecture and training configuration used for HAM10000 without modification. The overall performance results are presented in Table 13 and the training convergence curves are shown in Figure 13. The proposed ensemble achieved an accuracy of 91.47%, a weighted F1-score of 91.28%, and a macro AUC of 0.9818 on the ISIC 2019 dataset, demonstrating strong classification performance across all eight diagnostic categories. The confusion matrices and ROC curves for all models are presented in Figures 14, 15, respectively. The per-class performance results presented in Table 14 show that DF achieved a perfect precision of 100.00%, NV achieved an outstanding recall of 98.52%, and BCC achieved the highest AUC of 0.9967. SCC and AK showed comparatively lower recall values of 76.19% and 78.61% respectively, due to their visual similarity with adjacent lesion categories and limited training samples. It is noted that this ISIC 2019 model is trained independently of the HAM10000 model, using the same architecture and training configuration applied to a separate dataset. These results therefore demonstrate that the proposed ensemble methodology generalizes to an independent dataset, rather than serving as an external validation of the specific HAM10000 model (see Section 4.14 for further discussion).

**Table 13:** Overall performance of the proposed ensemble framework on the ISIC 2019 dataset.

Metric	Value
Accuracy	91.47%
Precision	91.54%
Recall	91.47%
F1 Score	91.28%
Macro AUC	0.9818

**Table 14:** Per-class performance of the proposed ensemble framework on the ISIC 2019 dataset.

Class	Precision (%)	Recall (%)	F1 (%)	AUC
AK	91.28	78.61	84.47	0.9754
BCC	91.65	94.14	92.88	**0.9967**
BKL	91.26	81.52	86.12	0.9878
DF	**100.00**	81.25	89.66	0.9748
MEL	92.61	80.42	86.09	0.9790
NV	90.93	**98.52**	**94.58**	0.9871
SCC	91.43	76.19	83.12	0.9675
VASC	**97.87**	90.20	93.88	0.9861

Bold values indicate the best performance for each metric.

### 4.14 Comparative analysis: HAM10000 and ISIC 2019

Table 15 presents a direct comparison of the proposed ensemble framework on both HAM10000 and ISIC 2019 datasets. The framework achieved 96.37% accuracy on HAM10000 and 91.47% on ISIC 2019. The approximately 5% performance difference is expected for three main reasons: first, ISIC 2019 is 2.5 times larger than HAM10000; second, ISIC 2019 contains eight classes compared to seven in HAM10000; and third, the two datasets differ in image acquisition protocols and lesion appearance distributions. Despite these differences, the framework maintains strong macro AUC values of 0.983 and 0.9818 on HAM10000 and ISIC 2019, respectively, indicating that the proposed methodology generalizes across different dermoscopic datasets.

**Table 15:** Performance comparison of the proposed ensemble framework on HAM10000 and ISIC 2019 datasets.

Metric	HAM10000	ISIC 2019
Accuracy	**96.37%**	91.47%
Precision	**96.25%**	91.54%
Recall	**96.37%**	91.47%
F1 Score	**96.23%**	91.28%
Macro AUC	**0.983**	0.9818
Classes	7	8
Images	10,015	25,331

Bold values indicate the best performance for each metric.

The results obtained on both datasets suggest that the proposed ensemble methodology is applicable across different dermoscopic image collections. The ISIC 2019 model is separately trained on an independent dataset, and these results demonstrate methodological generalizability rather than external validation of the specific HAM10000 model or its reported 96.37% accuracy. The strong performance on ISIC 2019, despite its larger scale and additional diagnostic category, demonstrates that the combination of heterogeneous ensemble fusion, Focal Loss, and class-specific augmentation effectively handles class imbalance and inter-class variability across diverse datasets. Furthermore, the consistently high AUC values across both datasets indicate the proposed framework's strong discriminative capability regardless of dataset characteristics.

It is important to acknowledge a key limitation of this study. The HAM10000 performance metrics reported (96.37% accuracy, 96.23% F1-score) are computed on the same 20% validation split used for early stopping and ensemble weight selection. We did not use an independently held-out HAM10000 test set, and we selected the weight configuration in Table 11 based on performance on this same split. Consequently, these figures should be interpreted as optimistic, tuned estimates rather than unbiased measures of generalization performance. The ISIC 2019 experiment (91.47% accuracy) uses a separately trained model on an independent dataset and demonstrates that the proposed ensemble methodology generalizes beyond HAM10000; however, it does not serve as an external test of the specific HAM10000 model and does not validate the 96.37% figure itself. A strict three-way split (training, validation, and held-out test) with k-fold cross-validation on HAM10000 is essential future work to obtain an unbiased estimate of the proposed framework's performance on this dataset.

### 4.15 Computational complexity and deployment considerations

The proposed ensemble framework combines three deep learning models: EfficientNet-B3, DenseNet201, and ResNet101. This improves classification performance and increases computational cost during training. The model was trained on an NVIDIA Tesla T4 GPU over 50 epochs using a batch size of 32. Training on the HAM10000 dataset took approximately 5 h, while training on the larger ISIC 2019 dataset took approximately 7 h using the same hardware setup. Although these training times are suitable for offline analysis, real-time clinical applications may require faster execution. To improve efficiency, several optimization methods can be considered. Techniques such as model pruning and quantization can reduce model size and inference time by removing unnecessary parameters and using lower-precision weight representations while maintaining performance. Knowledge distillation can transfer knowledge from the heavyweight ensemble to a lightweight model suitable for devices with limited computational resources. Another approach is to use an early exit mechanism, where predictions are made as soon as a high confidence level is achieved, without processing through all three models. These optimization techniques can help in reducing the computational requirements and are promising directions for future work to support the practical use of the proposed framework in a real-time environment.

## 5 Conclusion

The study proposed a weighted soft voting ensemble architecture consisting of three heterogeneous deep learning models, EfficientNet-B3, ResNet101, and DenseNet201, for automated skin lesion classification on the HAM10000 dataset. The framework integrated class-specific augmentation, focal loss weighting, and multi-method explainability validation to address the dual challenges of class imbalance and clinical trustworthiness, effectively ensuring optimized accuracy, transparency, and interpretability. The proposed ensemble framework fulfilled all the study objectives. First, the framework achieved an overall accuracy of 96.37%, precision of 96.25%, recall of 96.37%, an F1-score of 96.23%, and a macro-average AUC of 0.983, exceeding the best-performing individual model, EfficientNet-B3 (95.38%), by 0.99 percentage points. Furthermore, it surpassed the clinically recommended AUC threshold of 0.95 for computer-aided diagnosis systems. Second, the model achieved perfect classification for BCC (100%, 35/35; 95% CI: 90.1%–100.0%) and VASC (100%, 13/13; 95% CI: 77.2%–100.0%), and an accuracy of 99.77% for MEL, ensuring reliable detection of clinically critical malignant lesions. Thirdly, the implementation of class-weighted Focal Loss (α = 1, γ = 2.2) integrated with class-specific augmentation facilitated the mitigation of majority loss dominance, wherein minority classes AKIEC, DF, and VASC maintained identifiable feature clusters in t-SNE visualization despite severe sample imbalance. Finally, to the best of our knowledge, this study is the first approach to validating ensemble explainability on HAM10000 using Grad-CAM, LIME, and Occlusion Sensitivity Analysis in a TMAI-based framework. Notably, all methods consistently generated positive scores, indicating that the framework's predictions are based on clinically significant lesion regions rather than background artifacts, increasing confidence among healthcare practitioners. To further assess the generalizability of

the proposed methodology, we conducted experiments on the ISIC 2019 benchmark dataset using a separately trained model. The framework achieved an accuracy of 91.47%, a weighted F1-score of 91.28%, and a macro-average AUC of 0.9818 without any architectural modifications, indicating that the proposed ensemble methodology is adaptable across datasets of varying scale and complexity. Furthermore, a 3-fold cross-validation conducted on the HAM10000 dataset with strict lesion-level partitioning yielded a mean accuracy of 95.56% ± 0.32% and a mean AUC of 0.9782 ± 0.0027, confirming the consistency and reliability of the reported results across different data partitions. The ISIC 2019 results demonstrate that the proposed methodology generalizes to an independent dataset but do not constitute external validation of the specific HAM10000 model. Although the proposed framework demonstrates prominent clinical potential, it requires additional validation on independent, multi-institutional datasets to justify its generalized implementation across diverse patient demographics and imaging equipment. The MEL-NV boundary, a well-known challenge even for expert dermatologists, represents a plausible future research direction, incorporating attention-based mechanisms and complementary metadata. Future research will emphasize transformer-based architectures for enhanced global feature learning and federated learning frameworks to enable privacy-preserving, multi-institutional model development. In association with this, machine unlearning techniques will be investigated to facilitate selective removal of specific patient data from trained models, eliminating the need for full retraining, thereby addressing patient privacy rights and GDPR compliance requirements critical for real-time clinical deployment. Evaluation metrics for validating unlearning efficiency will include forget quality, model utility retention, and resistance to membership inference attacks. It will quantify the effectiveness of data removal while preserving diagnostic performance. Furthermore, the framework will be optimized for real-time clinical deployment by applying model compression and quantization techniques.

## Data Availability

The original contributions presented in the study are included in the article/supplementary material, further inquiries can be directed to the corresponding author.
